# Tuning Uranium Redox Chemistry in Asymmetric Polyoxometalate Complexes: Access to U(IV), U(V), and Transient U(VI)

**DOI:** 10.1021/acs.inorgchem.6c01673

**Published:** 2026-06-11

**Authors:** Dominic Shiels, William W. Brennessel, Ellen M. Matson

**Affiliations:** Department of Chemistry, 6927 University of Rochester, Rochester, New York 14627, United States

## Abstract

We report the synthesis and characterization of asymmetric sandwich-type complexes [TpU^IV^EW_11_O_39_]^x–^ (E = P; *x* = 4, E = Si; *x* = 5, Tp = trispyrazolylborate). These complexes represent rare examples of the selective formation of asymmetric actinide-polyoxometalate (An-POM) complexes. Oxidation studies show that the U^V^ analogues are stable and accessible, with both complexes isolated. Attempts to access U^VI^ with this framework yield mixed results. Attempts to produce [TpU^VI^PW_11_O_39_]^2–^ only led to decomposition, while electrochemical oxidation experiments gave evidence of transient formation of [TpU^VI^SiW_11_O_39_]^3–^ in solution. Rapid decomposition of [TpU^VI^SiW_11_O_39_]^3–^ inhibits full characterization. These studies indicate that the redox properties and stability of An-POM complexes containing actinides in higher valencies are dependent on the anionic charge of the assembly.

## Graphical abstract

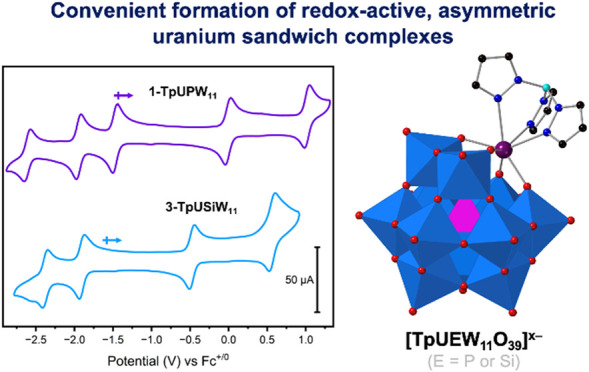

## Introduction

Investigating the coordination chemistry of actinides is an active area of research aimed at understanding the role of valence 5f orbitals in bonding toward the development of chemical approaches for the selective sequestration of radiological impurities from nuclear waste streams.^1–3^ This is of particular interest for the early actinides, where the relatively large radial extension of the valence 5f orbitals facilitates overlap with ligand-based orbitals, and thus bonds can have non-negligible f-character.^4,5^ Furthermore, the small energy differences between the 7s, 6d, and 5f orbitals in early actinides lead to a range of accessible oxidation states (An^II^-An^VII^).^5–8^ These factors combine to differentiate the coordination chemistry of early actinides from both lanthanides and late actinides, whose chemistries are dominated by the 3+ oxidation state.^9^ Understanding these differences in redox properties and bonding can facilitate the development of ligands that selectively bind early actinides, in a range of oxidation states, and mediate their separation from complex mixtures of waste products.^2,3,10,11^

One way to develop an understanding of how changes in oxidation state, and thus f-electron count, influence bonding in actinides is to develop an isostructural series of complexes that differ only in the oxidation state of the actinide center. In such systems, changes in bonding can be systematically evaluated as a function of the An oxidation state, which modulates factors including the ionic radius of An center, the radial extension and energies of the valence 5f orbitals, crystal-field splitting, and spin–orbit coupling.^12–14^ In this context, uranium is extremely interesting as it can readily access five unique redox states (U^II^–U^VI^).^15–18^ Though coordination complexes have been reported across this range of uranium valencies, there are only a few examples of complexes that stabilize uranium in more than two valencies due to the distinct bonding preferences of disparate oxidation states of uranium. Meyer leveraged acetylacetonate (acac) ligands to produce a series of square antiprismatic U^III/IV/V^ complexes, with electron paramagnetic resonance spectroscopy (EPR) highlighting the changes in magnetic properties across the series.^19^ The groups of Meyer, Huang, Hayton, and La Pierre have also successfully stabilized a series of U^IV/V/VI^ complexes using contrasting approaches.^20–22^ The groups of Meyer and Huang harnessed multidentate cyclen or arene-anchored ligands to stabilize uranium across three valencies, while Hayton and La Pierre exploited alkoxide and imidophosphorane ligands, respectively, to isolate homoleptic actinide complexes capable of undergoing one or two electron oxidations without significant structural change.^20–24^

In recent years, polyoxometalates (POMs), which are anionic molecular metal oxide clusters typically based on W^VI^, Mo^VI^, or V^V^, have become an increasingly popular platform for the investigation of actinide coordination chemistry. Interest in these early transition metal oxide assemblies is credited to the following: (1) POMs possess high molecular weights (ca. 1000 to 10,000 g mol^–1^), limiting the quantity of radioactive material required for stoichiometric reactions; (2) POMs are chemically and radiolytically robust, typically being oxidatively stable and freely manipulated in the presence of air and/or water; (3) An-POM complexes readily crystallize, providing access to precise structural metrics via single-crystal X-ray diffraction (SCXRD) methods; and (4) series of structurally similar An-POM complexes can be produced, allowing direct comparison of the influence of the actinide on the electronic properties of the complex.^25–34^ There has been a renaissance in An-POM chemistry in recent years, with the aqueous methods previously used to isolate Th-, U-, and Np-POM complexes being extended to heavier actinides, including Pu, Am, Cm, and Cf (examples of common structures are shown in Figure 1).^35–45^ Unfortunately, working with these systems in aqueous media limits electrochemical investigations, with reports examining the redox chemistry of An-POM complexes scarce.^46^ Looking specifically at uranium, all U-POM complexes synthesized in aqueous media feature either U^IV^ or [U^VI^O_2_]^2+^, with both U^V^ and “non-uranyl” U^VI^ being unknown.^47–54^

**Figure 1:**
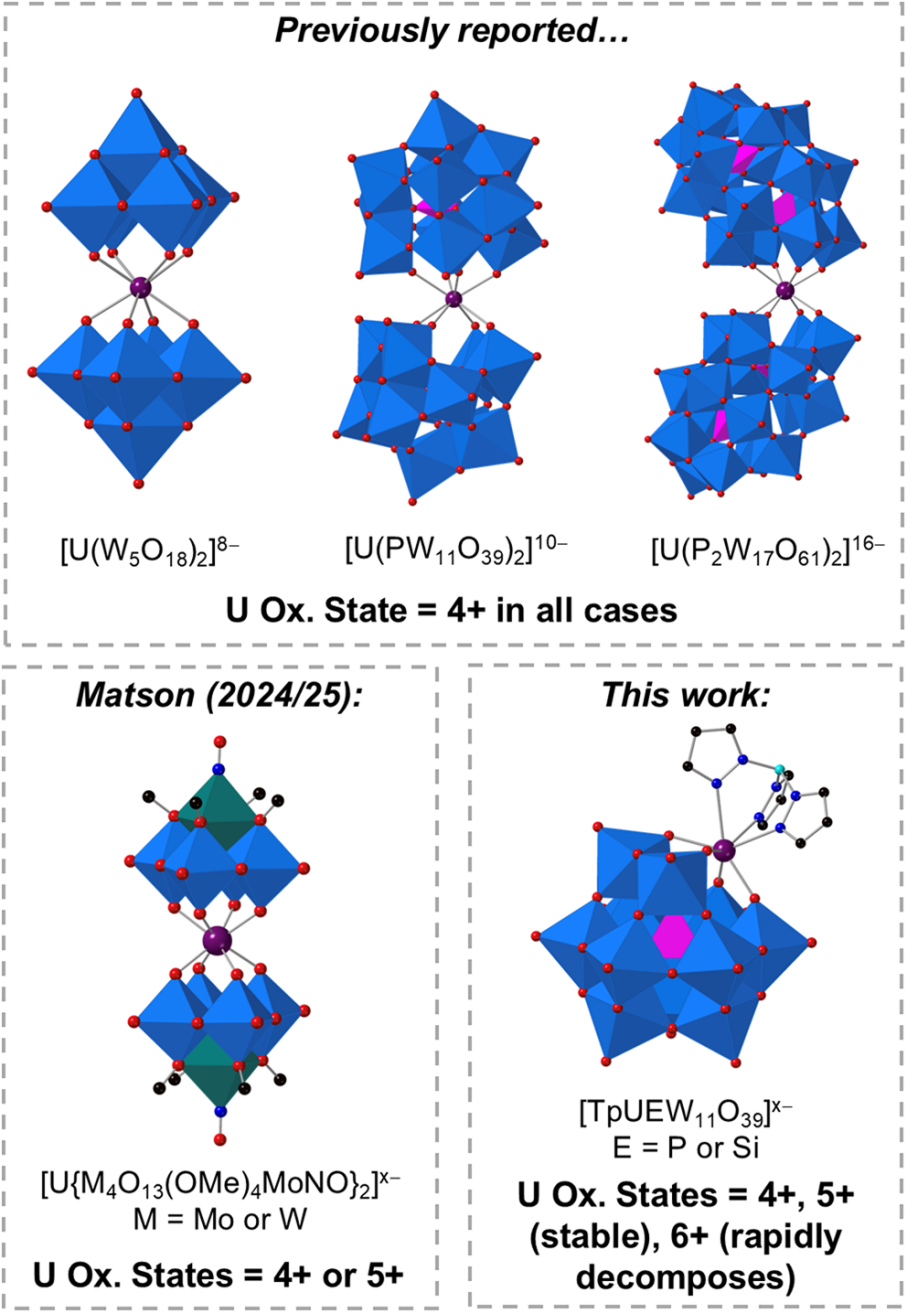
Overview of some of the previously reported aqueous U-POM chemistry and how it compares to recent nonaqueous U-POM chemistry reported by our group in terms of accessible uranium valencies.

Recent advances in nonaqueous POM chemistry have demonstrated that lacunary POM frameworks can be manipulated under organic conditions to enable controlled incorporation of metal centers and access to reactivity not available in aqueous media.^55–57^ Previously, our group has utilized preformed lacunary polyoxoalkoxide metalloligands, (TBA)_2_[M_4_O_13_(OMe)_4_MoNO]­[Na­(MeOH)] (M = Mo and W), to produce actinide-centered sandwich-type complexes with the general formula (TBA)_2_[An^IV^{M_4_O_13_(OMe)_4_MoNO}_2_] (An = Th, U, and Np).^58–60^ The uranium-containing analogues are listed in Figure 1. These complexes represent rare examples of An-POM complexes that were synthesized and are soluble in organic media. Analysis of the redox properties of the uranium-containing complexes by cyclic voltammetry reveals the presence of a reversible U^V/IV^ redox couple at ca. 0.75 V vs Fc^+/0^, suggesting that the U^V^-containing analogues of these complexes should be accessible. One-electron oxidation of the U^IV^ sandwich-type complexes by treatment with [NO]­[PF_6_] results in isolation of (TBA)­[U^V^{M_4_O_13_(OMe)_4_MoNO}_2_] (M = Mo or W).^58–60^ These complexes are isostructural to their U^IV^ congeners and represent the first examples of U^V^–POM complexes to be isolated and fully characterized. The electronic properties of the complexes vary as a function of uranium oxidation state, giving valuable insights into how the interactions between the uranium center and the metalloligand change as a function of actinide valency.^58–60^ Unfortunately, electrochemical analysis of these complexes shows no evidence of an accessible U^VI/V^ redox couple. Instead, the sandwich-type complexes undergo irreversible oxidation at ca. 1.35 V vs Fc^+/0^, attributed to oxidation of the {Mo-NO}^4^ unit of the metalloligand and subsequent decomposition of the POM.^58–60^ These factors combine to prohibit access to the isostructural U^VI^ complexes, limiting comparative studies across only two oxidation states of the actinide center (i.e., U^IV^ and U^V^).

Interested in developing an alternative approach that would allow a single POM platform to stabilize U^IV^, U^V^, and U^VI^, we hypothesized that replacing this polyoxoalkoxide ligand with an all-inorganic, more anionic POM would both stabilize higher uranium valencies and increase the oxidative stability of the assembly. This in turn would allow access to “non-uranyl” U^VI^ adducts of metal oxide assemblies. Lacunary Keggin-type POMs, with the general formula [XW_11_O_39_]^n–^ (X = B, Si, P, Ge, Ga), are known to be oxidatively stable and thus could fill this role.^61–64^ While the anionic charge and facile coordination to uranium render these POMs as an intriguing starting point for our investigations, it is challenging to translate this chemistry (i.e., U­(POM)_2_) into an organic solvent to create an environment where “non-uranyl” U^V^ and U^VI^ complexes are stable. This is due to the fact that the cations required to solubilize lacunary Keggin precursors in organic solvents (typically quaternary ammonium cations like TBA^+^) complicate purification of the materials when charges of the overall assembly become too high.

Herein, we describe a hybrid approach, in which a single lacunary Keggin POM (either [PW_11_O_39_]^7–^ or [SiW_11_O_39_]^8–^) forms half of a uranium-centered sandwich-type complex, while the classic multidentate organometallic ligand, trispyrazolylborate, [HB­(C_3_N_2_H_3_)_3_]^−^ (Tp), occupies the opposite face (Figure 1). Combining the two allows us to reduce the overall anionic charge of the system when compared to classical U­(POM)_2_ systems,^25,47,48,54^ to find a balance between ease of synthesis/purification and U^V^/U^VI^ stability. We first synthesized and characterized the U^IV^ complex (TBA)_4_[TpUPW_11_O_39_]. Electrochemical analysis reveals the presence of reversible U^V/IV^ and U^VI/V^ redox couples. Oxidation studies revealed that the complex can be oxidized by one electron to (TBA)_3_[TpU^V^PW_11_O_39_]; however, further oxidation to U^VI^ results in decomposition of the assembly. We hypothesized that we could further stabilize the higher uranium valencies by increasing the anionic charge on the assembly, motivating the synthesis and characterization of (TBA)_5_[TpUSiW_11_O_39_]. Cyclic voltammetry experiments reveal that the U-based redox events shift cathodically when compared to corresponding events in the CV of (TBA)_4_[TpUPW_11_O_39_], which is attributed to the higher anionic charge of the assembly. As expected, oxidation to U^V^ was successful, with characterization data being almost identical to that of (TBA)_3_[TpU^V^PW_11_O_39_]. Oxidation experiments targeting (TBA)_3_[TpU^VI^SiW_11_O_39_] monitored by UV–vis/NIR and ^1^H NMR spectroscopy suggest the target complex does form in solution; however, it is unstable, decomposing within 1 h at room temperature. Though we have been unable to isolate and fully characterize a U^VI^ complex, we have demonstrated that U-based redox properties and the stability of U^V^/U^VI^ in POM systems are charge dependent.

## Experimental Section

### General Considerations

Air- and moisture-sensitive manipulations with all complexes were carried out using a standard high-vacuum line, Schlenk techniques, or an MBraun inert atmosphere drybox containing an atmosphere of purified dinitrogen. The MBraun glovebox was equipped with a cold well designed for freezing samples in liquid nitrogen, as well as a −35 °C freezer for cooling samples and crystallizations. Solvents for sensitive manipulations were dried and deoxygenated using literature procedures with a Seca solvent purification system or a glass contour solvent purification system (Pure Process Technology, LLC) and stored over activated 4 Å molecular sieves (Fisher Scientific) prior to use. Deuterated solvents were purchased from Cambridge Isotope Laboratories, dried with molecular sieves, and degassed by three freeze–pump–thaw cycles. Na_3_[PW_12_O_40_]·8H_2_O,^65^ UCl_4_,^66^ K_8_[SiW_11_O_39_]·13H_2_O,^67^ (TBA)_4_H_4_[SiW_11_O_39_],^68^ TBA­(Tp),^69^ and U­[{N­(SiMe_3_)­(SiMe_2_CH_2_)}­{N­(SiMe_3_)_2_}_2_][Bibr ref70] were synthesized using previously reported methods. Tetrabutylammonium hexafluorophosphate (TBAPF_6_) was purchased from Oakwood Chemical and recrystallized three times from hot ethanol before use. All other reagents were purchased from commercial sources (Fisher Scientific, VWR, and MilliporeSigma) and used without further purification.

### Safety Considerations

**Caution!** *Depleted uranium (primary isotope* ^238^ *U) is a weak α-emitter (4.197 MeV) with a half-life of 4.47 × 10* ^9^ *years; manipulations and reactions should be carried out in monitored fume hoods or in an inert atmosphere drybox in a radiation laboratory equipped with α and β counting equipment.*

### Synthesis of (TBA)_6_[NaPW_11_O_39_]

This procedure is adapted from the method originally described by Errington and co-workers.^71^ In a 150 mL Schlenk flask, Na_3_[PW_12_O_40_]·8H_2_O (10 g, 3.2 mmol, 1 equiv) was suspended in MeCN (30 mL). To this stirring suspension, TBA­(OH) (13.9 mL, 19.4 mmol, 1.4 M in MeOH, 6 equiv) was added. The mixture was stirred at room temperature for 16 h. The suspension was transferred to a series of 10 mL centrifuge tubes, where the fine white precipitate was separated from the solution by centrifugation. The supernatant solution was collected, and the solvent was removed under reduced pressure. This led to the formation of a thick oil or tacky solid. The product was taken up in DCM (50 mL), and the solution was filtered to remove any residual solids. DCM was removed under reduced pressure, and the resulting solid was triturated with Et_2_O until a free-flowing powder was obtained. Drying the resultant powder under vacuum gave 11.1 g (83% yield) of crude material, which was used without further purification. The product is hygroscopic and should be stored under an inert atmosphere. ^1^H NMR (500 MHz, CD_3_CN) δ, 0.97 (t, *J* = 7.4 Hz, CH_3_, 72 H), 1.38 (h, *J* = 7.4 Hz, CH_2_, 48 H), 1.63 (m, CH_2_, 48 H), 3.17 (m, CH_2_, 48 H). ^31^P NMR (202.4 MHz, CD_3_CN) δ −9.80 ppm.

### Synthesis of (TBA)_4_[TpU^IV^PW_11_O_39_] (1-TpUPW_11_)

In separate vials, UCl_4_ (0.023 g, 0.06 mmol) and KTp (0.015 g, 0.06 mmol) were dissolved in THF (2 mL). The KTp solution was added to the UCl_4_ solution, and the mixture was stirred for 15 min at room temperature, during which time a cloudy green solution formed. In a separate vial, (TBA)_6_[NaPW_11_O_39_] (0.250 g, 0.06 mmol) was dissolved in MeCN (4 mL). The solution containing the POM was added rapidly to the “TpUCl_3_” solution. An instantaneous color change to red/brown is observed. The solution was stirred for an additional 30 min at room temperature before the solvent was removed under vacuum. The tacky red/brown residue was dissolved in DCM (2 mL). Et_2_O (8 mL) was added quickly with stirring, resulting in the formation of a red/brown suspension. The mixture was allowed to settle, and the DCM/Et_2_O mixture was decanted. The solid was then sequentially triturated with DCM/Et_2_O (1:2, 2 × 5 mL), THF (2 × 5 mL), and Et_2_O (5 mL). The solid was then dissolved in DCM (20–30 mL), and the resulting slightly cloudy red/brown solution was filtered through a bed of Celite (ca. 1 cm). The solvent was then removed under vacuum to leave a dark red solid (0.186 g, 75% yield). The crude material was crystallized by vapor diffusion of Et_2_O into a saturated solution of the complex dissolved in MeCN, giving dark orange/red blocks (0.110 g, 45% yield). ^1^H NMR (500 MHz, CD_3_CN) δ, −25.74 (B–H, 1 H), −4.06 (ArH, 3 H), 1.19 (TBA, 48 H), 1.67 (TBA, 32 H), 1.88 (TBA, 32 H), 3.38 (TBA, 32 H), 7.08 (ArH, 3 H), 44.14 (ArH, 3 H). ^31^P NMR (202.4 MHz, CD_3_CN) δ −124.11 ppm. UV–vis/NIR (21 °C, MeCN): λ_max_ (ε) = 478 nm (533 M^–1^ cm^–1^), 684 nm (176 M^–1^ cm^–1^), 1096 nm (96 M^–1^ cm^–1^), 1142 nm (99 M^–1^ cm^–1^), 1172 nm (104 M^–1^ cm^–1^), 1376 nm (42 M^–1^ cm^–1^), 1470 nm (42 M^–1^ cm^–1^) (+ intense absorption at <400 nm). Anal. calcd for C_73_H_154_N_10_O_39_BPW_11_U: C, 21.40%; H, 3.79%; N, 3.42%. Found: C, 21.36%; H, 3.90%; N, 3.46%.

### Synthesis of (TBA)_3_[TpU^V^PW_11_O_39_] (2-TpU^V^PW_11_)

In a 20 mL scintillation vial, [N­(C_6_H_4_Br-4)_3_]­[SbCl_6_] (0.033 g, 0.040 mmol, 1.1 equiv) was dissolved in MeCN (2 mL). A solution of (TBA)_4_[TpU^IV^PW_11_O_39_] (0.150 g, 0.037 mmol, 1 equiv) dissolved in MeCN (2 mL) was added to the solution. The solution was stirred for 30 min during which time a color change from red/brown to orange/brown was observed. The solution was then filtered through a bed of Celite (ca. 1 cm). The eluent was collected, and the solvent was removed under reduced pressure to yield a tacky orange solid. The solid was then triturated with DCM/Et_2_O (1:1, 2 × 5 mL) and Et_2_O (10 mL) to give a free-flowing orange powder. After drying under vacuum, the crude product (0.109 g, 77% yield) was crystallized by vapor diffusion of Et_2_O into a saturated solution of the product in DMF to give 0.084 g (60% yield) of orange crystals. ^1^H NMR (500 MHz, CD_3_CN) δ, −8.55 (B–H, 1 H), 1.02 (TBA, 36 H), 1.43 (TBA, 24 H), 1.68 (TBA, 24 H), 2.90 (ArH, 3 H), 3.17 (TBA, 24 H), 6.94 (ArH, 3 H), 24.19 (ArH, 3 H). ^31^P NMR (202.4 MHz, CD_3_CN) δ −80.71 ppm. UV–vis/NIR (21 °C, MeCN): λ_max_ (ε) = 1050 nm (54 M^–1^ cm^–1^), 1352 nm (44 M^–1^ cm^–1^), 1586 nm (126 M^–1^ cm^–1^) (+ intense absorption features that onset at ca. 600 nm and steadily rise when scanning to higher energies). Anal. calcd for C_57_H_118_N_9_O_39_BPW_11_U: C, 17.81%; H, 3.09%; N, 3.28%. Found: C, 18.01%; H, 3.04%; N, 3.13%.

### Synthesis of (TBA)_5_[TpU^IV^SiW_11_O_39_] (3-TpUSiW_11_)

In a 20 mL scintillation vial, (TBA)_4_H_4_[SiW_11_O_39_] (0.300 g, 0.08 mmol) was dissolved in MeCN (4 mL). A solution of TBA­(Tp) (0.038 g, 0.08 mmol) dissolved in MeCN (1 mL) was added. To this stirring solution, a solution of U­[{N­(SiMe_3_)­(SiMe_2_CH_2_)}­{N­(SiMe_3_)_2_}_2_] (0.059 g, 0.08 mmol) in THF (1 mL) was added. The mixture immediately turned dark red/brown. The mixture was stirred for 30 min at room temperature before the solvent was removed under reduced pressure. The tacky red/brown residue was dissolved in DCM (1 mL). Et_2_O (12 mL) was added quickly with stirring to give a red/brown suspension. The mixture was allowed to settle, and the DCM/Et_2_O mixture was decanted. This process was repeated once more. The solid was then sequentially triturated with THF (5 mL) and Et_2_O (5 mL). The solid was then dried under vacuum to leave a brown/red powder (0.212 g, 59% yield). The crude material was crystallized by vapor diffusion of Et_2_O into a saturated solution of the complex dissolved in MeCN/DMF, giving dark brown blocks (0.153 g, 43% yield). ^1^H NMR (500 MHz, CD_3_CN) δ, −26.98 (B–H, 1 H), −4.76 (ArH, 3 H), 1.17 (TBA, 60 H), 1.68 (TBA, 40 H), 1.89 (TBA, 40 H), 3.45 (TBA, 40 H), 6.31 (ArH, 3 H), 45.05 (ArH, 3 H) (note: CD_3_CN reference signal was obscured by a TBA resonance and therefore chemical shifts may vary to a small degree). UV–vis/NIR (21 °C, MeCN): λ_max_ (ε) = 478 nm (519 M^–1^ cm^–1^), 682 nm (218 M^–1^ cm^–1^), 1092 nm (101 M^–1^ cm^–1^), 1124 nm (117 M^–1^ cm^–1^), 1150 nm (114 M^–1^ cm^–1^), 1352 nm (56 M^–1^ cm^–1^), 1382 nm (60 M^–1^ cm^–1^), 1460 nm (64 M^–1^ cm^–1^) (+ intense absorption at <400 nm). Anal. calcd for C_73_H_154_N_10_O_39_BSiW_11_U·3DMF·Et_2_O: C, 26.45%; H 4.81%; N, 4.23%. Found: C, 26.74%; H, 4.44%; N, 4.36%.

### Synthesis of (TBA)_4_[TpU^V^SiW_11_O_39_] (4-TpU^V^SiW_11_)

In a 20 mL scintillation vial, (TBA)_5_[TpUSiW_11_O_39_] (0.229 g, 0.053 mmol) was dissolved in MeCN (4 mL), resulting in a dark brown solution. This was then added to a separate vial containing solid Fc­[PF_6_] (0.18 g, 0.054 mmol) with stirring. The mixture was stirred for 15 min during which time the solution turned from dark brown to orange. The volatiles were removed under reduced pressure to yield a tacky orange solid. Trituration with Et_2_O (2 × 5 mL) allowed removal of Fc (with washings appearing yellow). The orange solid was then further triturated with DCM:Et_2_O (1:1, 2 × 5 mL) and Et_2_O (2 × 5 mL). The resulting orange powder was then dried under vacuum. The crude product (0.141 g, 65% yield) was crystallized by vapor diffusion of Et_2_O into a saturated solution of the product in MeCN/DMF, giving orange crystals (0.117 g, 54% yield). ^1^H NMR (500 MHz, CD_3_CN) δ, −4.03 (B–H, 1 H), 1.04 (TBA, 48 H), 1.48 (TBA, 32 H), 1.74 (TBA, 32 H), 3.26 (TBA, 32 H), 4.56 (ArH, 3 H), 6.29 (ArH, 3 H), 18.57 (ArH, 3 H). UV–vis/NIR (21 °C, MeCN): λ_max_ (ε) = 922 nm (31 M^–1^ cm^–1^), 1048 nm (42 M^–1^ cm^–1^), 1340 nm (34 M^–1^ cm^–1^), 1564 nm (170 M^–1^ cm^–1^) (+ intense absorption that onsets at ca. 600 nm and steadily rises when scanning to higher energies). Anal. calcd for C_73_H_154_N_10_O_39_BSiW_11_U: C, 21.41%; H, 3.79%; N, 3.42%. Found: C, 21.41%; H, 3.54%; N, 3.47%.

### Attempted Synthesis of (TBA)_3_[TpU^VI^SiW_11_O_39_]

In a 20 mL scintillation vial, (TBA)_4_[TpU^V^SiW_11_O_39_] (0.050 g, 0.012 mmol) was dissolved in MeCN (2 mL). This was added quickly to a separate vial containing [N­(C_6_H_4_Br-4)_3_]­[SbCl_6_] (0.010 g, 0.012 mmol or 0.020 g, 0.024 mmol) suspended in MeCN (2 mL). The resulting dark orange/brown mixture was stirred for 20 min before filtering through a bed of Celite (ca. 1 cm). The eluent was collected, and the solvent was removed under reduced pressure to yield a tacky orange solid. The solid was washed sequentially with Et_2_O (5 mL), DCM (5 mL), THF (5 mL), and then again with Et_2_O (5 mL). The orange solid was then dried under vacuum to give a free-flowing powder. ^1^H NMR (500 MHz, CD_3_CN) δ, 1.00 (TBA, 36 H), 1.42 (TBA, 24 H), 1.67 (TBA, 24 H), 3.17 (TBA, 24 H), 6.76 (ArH), 8.31 (ArH), 8.53 (ArH). UV–vis/NIR (21 °C, MeCN): Intense absorption that onsets at ca. 600 nm and steadily rises when scanning to higher energies (Note: Features associated with ***4-TpU*** ^***V***^ ***SiW***_***11***_ are also consistently observed). The limited stability of (TBA)_3_[TpU^VI^SiW_11_O_39_], combined with the inability to drive full conversion of starting material to product, inhibited our ability to characterize using elemental analysis.

### Physical Measurements

^1^H NMR spectra were recorded at room temperature on a 400 MHz Bruker AVANCE spectrometer or a 500 MHz Bruker AVANCE spectrometer locked to the signal of deuterated solvents. All chemical shifts are reported relative to tetramethylsilane by using the chosen deuterated solvent as a standard. Cyclic voltammetry was performed using a three-electrode setup inside a positive-pressure glovebox (MBraun UniLab, USA) using a Bio-Logic SP 150 potentiostat/galvanostat. The concentrations of the cluster and the supporting electrolyte (TBAPF_6_) were kept at 1 mM and 100 mM, respectively, throughout all measurements. Cyclic voltammograms (CVs) were recorded using a 3 mm diameter glassy carbon working electrode (CH Instruments, USA), a Pt wire auxiliary electrode (CH Instruments, USA), and a Ag/Ag^+^ nonaqueous reference electrode with 0.01 M AgNO_3_ in 0.1 M TBAPF_6_ in acetonitrile (BASi, USA). Ferrocene was used as an internal standard after completion of the measurements, and potentials were referenced versus the Fc^+/0^ couple. Electronic absorption measurements were recorded at room temperature in anhydrous MeCN in sealed 1 cm quartz cuvettes using an Agilent Cary 6000i UV–vis-NIR spectrophotometer. Elemental analysis data were obtained from the Elemental Analysis Facility at The University of Rochester. Microanalysis samples were weighed with a PerkinElmer model AD6000 autobalance, and their compositions were determined with a PerkinElmer 2400 series II analyzer. Air-sensitive samples were handled in a VAC Atmospheres glovebox.

### X-ray Crystallography

A single crystal of **1-TpUPW**_**11**_ was placed onto a nylon loop and mounted on a Rigaku XtaLAB Synergy-S Dualflex diffractometer equipped with a HyPix-6000HE HPC area detector for data collection at 100.00(10) K. A preliminary set of cell constants and an orientation matrix were calculated from a small sampling of reflections.^72^ A short pre-experiment was run, from which an optimal data collection strategy was determined. The full data collection was carried out using a PhotonJet (Mo) X-ray source. After the intensity data were corrected for absorption, the final cell constants were calculated from the xyz-centroids of the strong reflections from the actual data collection after integration.^72^ The structure was solved using SHELXT or isomorphic replacement and refined using SHELXL.^73,74^ Most or all non-hydrogen atoms were assigned from the solution. Full-matrix least-squares/difference Fourier cycles were performed, which located any remaining non-hydrogen atoms. All non-hydrogen atoms were refined with anisotropic displacement parameters. All hydrogen atoms were placed in ideal positions and refined as riding atoms with relative isotropic displacement parameters.

## Results and Discussion

### Synthesis and Characterization of (TBA)_4_[TpU^IV^PW_11_O_39_] ] (1-TpUPW_11_)

To prevent the formation of mixtures of sandwich-type complexes (i.e., {TpUPOM} + {Tp_2_U} + {U­{POM)_2_}), we hypothesized that preassembly of the organic ligand with the uranium precursor would increase selectivity for the asymmetric sandwich-type complex. Though TpUCl_3_ is unknown, there are two reports that show that KTp* (K­[HB­(3,5-Me_2_Pz)_3_]) reacts with UCl_4_ to give either Tp*UCl_3_ or Tp*UCl_3_(THF).^75,76^ We therefore first combined KTp with UCl_4_ in THF (Scheme 1), targeting *in situ* formation of “TpAnCl_3_” (though this intermediate was not characterized). To this mixture was then added the lacunary Keggin-type POM, (TBA)_6_[NaPW_11_O_39_] (Scheme 1).^71^ The reaction mixture immediately changed color from light green to dark red/brown upon addition of the POM. This observation is consistent with the generation of the target asymmetric sandwich-type complex (TBA)_4_[TpU^IV^PW_11_O_39_] (**1-TpUPW**_**11**_), as many U^IV^–POM complexes are reported to be brown in color.^28,52,53^ Following workup (see Experimental Section for details), product formation was confirmed by ^1^H NMR and ^31^P NMR spectroscopies ­(Figure 2). The ^1^H NMR spectrum contains four resonances in a 3:3:3:1 ratio, which are assigned to the 3 × CH groups of the pyrazole rings and the B–H unit of the Tp ligand (Figure 2, top**)**. Four additional intense signals are observed between 0 and 4 ppm, assigned to the four TBA cations that charge balance the anionic assembly. The chemical shifts of the resonances assigned to the Tp ligand (−26 and 45 ppm) are shifted compared to those of KTp^77^ due to its proximity to the paramagnetic actinide center (U^IV^, f^2^). The ^31^P NMR spectrum shows a single resonance, −124.11 ppm, again paramagnetically shifted from that of the starting material, (TBA)_6_[NaPW_11_O_39_] (−9.80 ppm). Collectively, these data provide strong evidence of the formation of the asymmetric sandwich-type complex.

**Figure 2:**
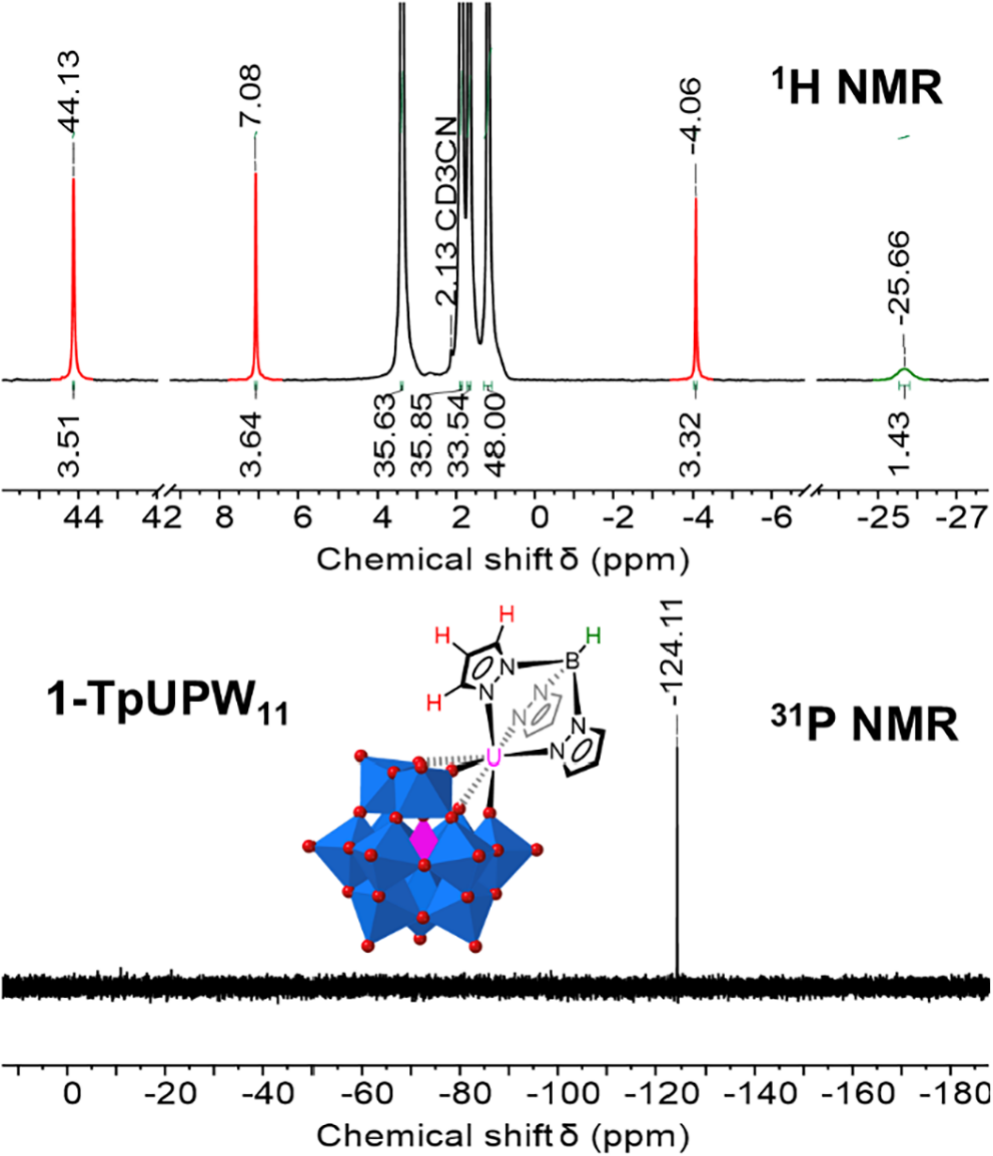
Obtained ^1^H NMR (top) and ^31^P NMR (bottom) spectra of **1-TpUPW**_**11**_. Spectra were obtained at room temperature in CD_3_CN. The proposed structure of **1-TpUPW**_**11**_ is also given, with the protons highlighted in red/green corresponding to the red/green peaks in the ^1^H NMR spectrum.

**Scheme 1:**
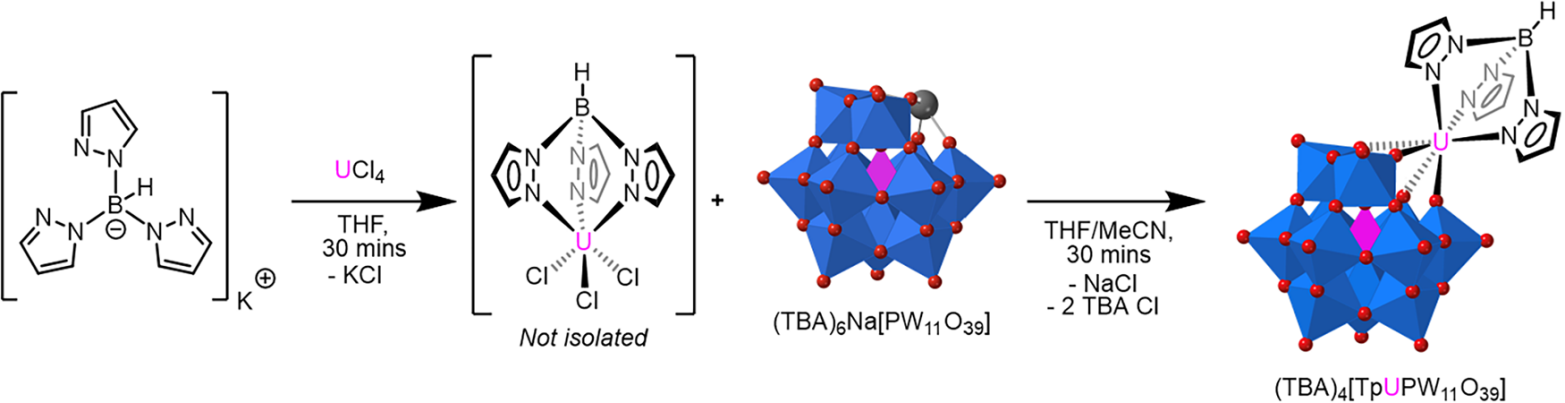
Synthetic Approach Used to Produce (TBA)_4_[TpUPW_11_O_39_] (**1-TpUPW**_**11**_)

To confirm the formation of the target complex, **1-TpUPW**_**11**_ was characterized by single-crystal X-ray diffraction (SCXRD). Red/brown single crystals suitable for analysis were obtained via vapor diffusion of Et_2_O into a concentrated solution of the complex dissolved in MeCN. After refinement of the data, the structure shown in Figure 3 was obtained, confirming the generation of the desired asymmetric sandwich-type complex. Complex **1-TpUPW**_**11**_ features a central seven-coordinate uranium ion. Analysis using Continuous Shape Measures (CShM) (Table S1) reveals that the coordination geometry at uranium is best described as capped octahedral (CShM value = 0.65), with one arm of the Tp ligand eclipsing the U–O15 bond (torsion angle = 1.9(5)°), while the other two are staggered between U–O bonds (torsion angles = 29.4(4)°, 31.4(5)°). The average U–N bond distances in **1-TpUPW**_**11**_ (ca. 2.53 Å) are slightly shorter than those reported in mono-Tp* uranium­(IV) complexes, consistent with the reduction in steric crowding associated with replacing Tp* with Tp.^75,76,78–81^ The average U–O distances (ca. 2.20 Å) are significantly shorter than those present in previously reported U­(POM)_2_ systems (typically 2.35–2.40 Å).^25,28,32,51,58,60^ This may be a consequence of the asymmetry of **1-TpUPW**_**11**_, where weaker interactions between the uranium center and the Tp ligand lead to tighter binding of the actinide to the anionic POM ligand. This is conveniently visualized by examining the distances between the uranium center and the respective N1–N3–N5 (1.90 Å) and O12–O13–O14-O15 (0.77 Å) planes (Figure 3).

**Figure 3:**
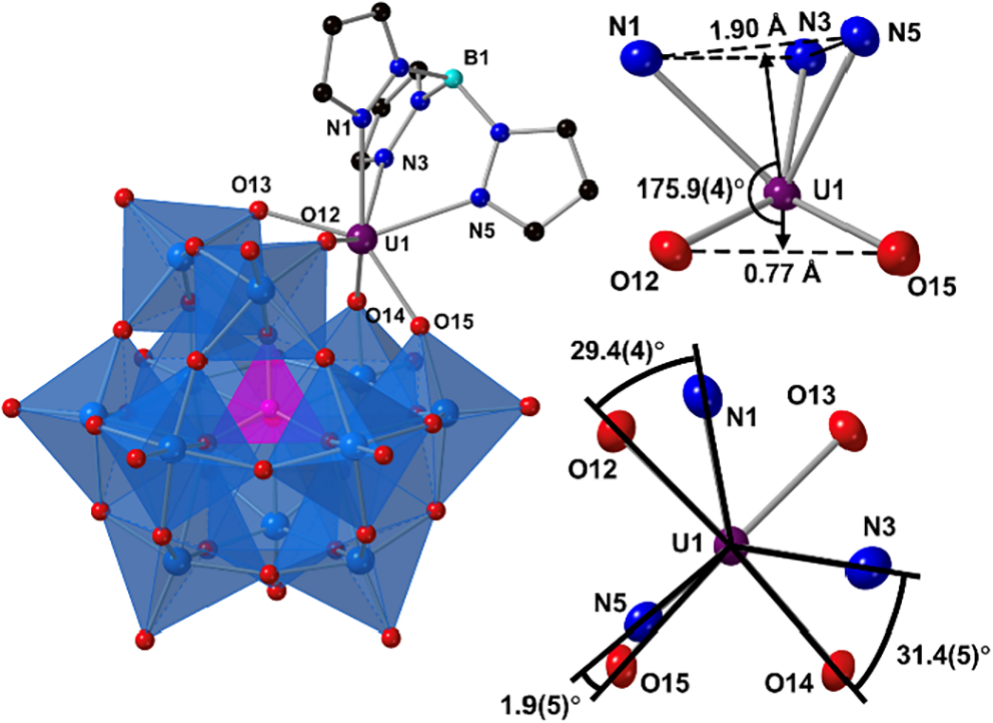
SCXRD structure of **1-TpUPW**_**11**_. Additional views of uranium and its interactions with surrounding donor atoms are given, highlighting relevant bond distances/angles (probability ellipsoids set at 50%). Cations and solvent molecules have been masked for clarity.

The electronic and vibrational properties of **1-TpUPW**_**11**_ were evaluated using electronic absorption (UV–vis/NIR) and infrared spectroscopies. The UV–vis/NIR spectrum of **1-TpUPW**_**11**_ (Figure 4) features several low-intensity transitions in the 700–1600 nm region (see Experimental Section for details). These peaks are commonly observed in UV–vis-NIR spectra of U^IV^ complexes and are attributed to transitions between the partially filled f-orbitals of U^IV^ (f^2^).^5,28,52,53^ Alongside these weak transitions, there is a more intense absorption observed at 478 nm (533 M^–1^ cm^–1^). This is caused by U^IV^(5f) → W^VI^(5d) (LUMO) charge transfer and is also present in the UV–vis-NIR spectra of many reported U^IV^–POM complexes.^28,52,53,59^ There is also more intense absorption observed at wavelengths shorter than 400 nm, which is likely caused by O­(2p) → W­(5d) ligand-to-metal charge transfer (LMCT) processes, but may also have contributions from O­(2p) → U­(5f) LMCT.^82–85^ Additional characterization by infrared spectroscopy (Figure S1) shows bands for ν­(BH) at 2463 cm^–1^, ν­(PO) at 1048 cm^–1^, ν­(W=O) at 951 cm^–1^, and ν­(WOW) at 789/769 cm^–1^, consistent with values for reported {TpM} and {M^IV^PW_11_O_39_}^4–^ complexes.^86–88^

**Figure 4:**
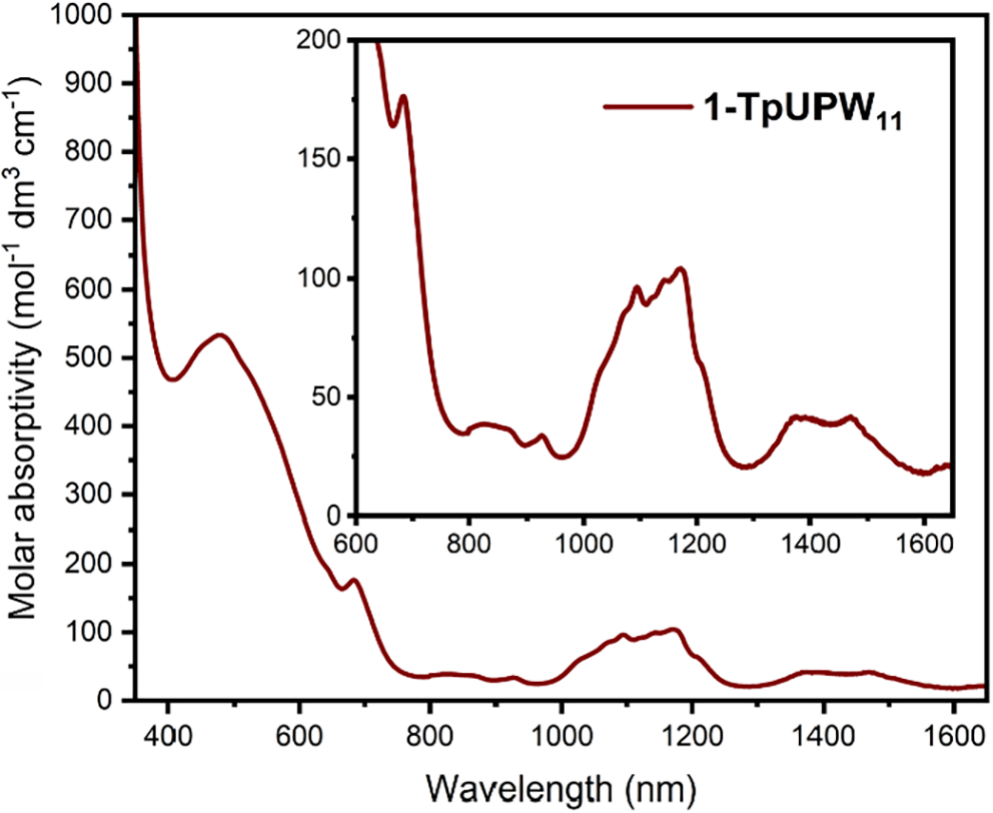
UV–vis-NIR spectrum of an MeCN solution of **1-TpUPW**_**11**_. The spectrum was obtained at 21 °C.

To examine the redox properties of **1-TpUPW**_**11**_, cyclic voltammetry was performed on this complex and compared to that of its lacunary precursor (TBA)_6_[NaPW_11_O_39_] (Figure 5). It is immediately apparent that the number of redox processes present in the CV of **1-TpUPW**_**11**_ far surpasses that of (TBA)_6_[NaPW_11_O_39_]. The CV of (TBA)_6_[NaPW_11_O_39_] features a single reduction event at −2.49 V vs Fc/Fc^+/0^; the extremely negative reduction potential is attributed to the high anionic charge of the lacunary POM. In contrast, the CV of **1-TpUPW**_**11**_ shows three reversible reduction events between ca. −1.5 to −2.8 V vs Fc^+/0^. We note that this behavior is comparable to the reported tungsten-based reduction events of (TBA)_4_[SiW_12_O_40_] in MeCN (*E*_1/2_ values range from −1 to −2.5 V vs Fc^+/0^).^89^ This illustrates that the reduction properties of Keggin-type POMs are correlated to the overall charge of the assembly in organic solvent, where lower charges of the assembly facilitate the reversible addition of more reducing equivalents within a set potential range.

**Figure 5:**
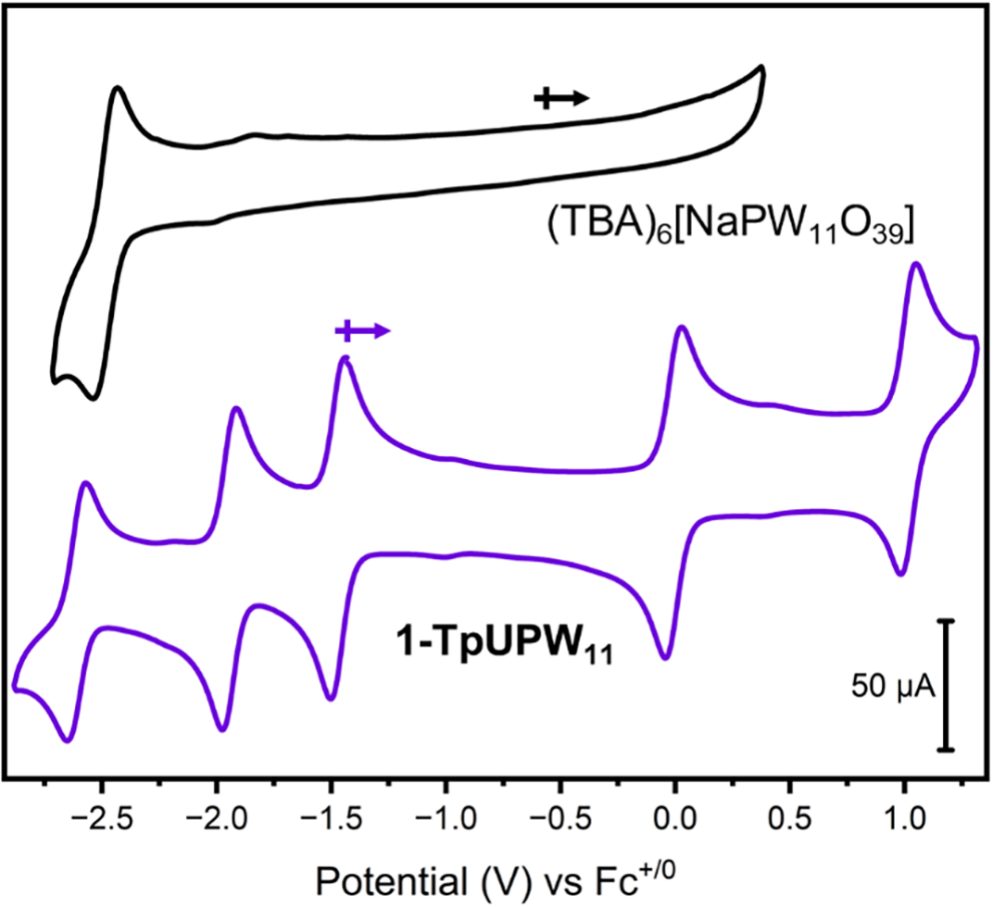
CVs of (TBA)_6_[NaPW_11_O_39_] and **1-TpUPW**_**11**_. CVs were obtained in a solution of 0.1 M TBA­(PF_6_) in MeCN at 200 mv/s. The analyte concentration in each experiment was 1 mM.

The CV of **1-TpUPW**_**11**_ also shows the presence of two reversible one-electron oxidation processes, located at +0.02 V and +1.02 V vs Fc^+/0^. The Tp ligand is known to be oxidatively inert, with no evidence of ligand oxidation observed in CVs of reported Tp complexes before ca. 1.5 V vs Fc^+/0^ in MeCN or DCM with TBA-based electrolytes.^90–95^ Furthermore, the POM metalloligand is bound in its highest oxidation state, as indicated by the lack of reversible oxidation processes in the CV of the starting material, and by the lack of intervalence charge transfer bands in the UV–vis (Figure S2). As such, the two oxidation events are assigned to the U^V/IV^ and U^VI/V^ redox couples, respectively. The U^V/IV^ redox couple of **1-TpUPW**_**11**_ occurs at a significantly lower potential than the corresponding redox couple in (TBA)_2_[U­{Mo_5_O_13_(OMe)_4_NO}_2_] (*E*_1/2_ = 0.74 V vs Fc^+/0^) or (TBA)_2_[U­{W_4_O_13_(OMe)_4_MoNO}_2_] (*E*_1/2_ = 0.76 V vs Fc^+/0^), suggesting that the Keggin-type POM is good at stabilizing high oxidation states of the actinide. Shifting of the U^V/IV^ redox couple to a much more modest potential subsequently allows the observation of the U^VI/V^ redox couple within the electrochemical window.

### Investigating the Oxidation of 1-TpUPW_11_

To probe the stability of the oxidized forms of **1-TpUPW**_**11**_, both electrochemical and chemical oxidation experiments were performed. The one-electron oxidation of **1-TpUPW**_**11**_ is achieved electrochemically by performing controlled potential electrolysis (CPE) at 0.23 V vs Fc^+/0^ on a red/brown solution of **1-TpUPW**_**11**_ dissolved in MeCN (with a supporting electrolyte). The solution gradually turns yellow/orange during the experiment. During CPE, 1.06 × 10^–5^ moles of electrons are passed (Figure S3), corresponding to the loss of approximately one electron per molecule of **1-TpUPW**_**11**_. After CPE, analysis of the solution by CV shows the presence of the same redox features, consistent with retention of the overall structure, but a change in the open-circuit potential (OCP), moving from −0.58 V vs Fc^+/0^ to 0.04 V vs Fc^+/0^ (Figure S4). This is consistent with the successful electrochemical formation of the U^V^ complex (TBA)_3_[TpU^V^PW_11_O_39_] (**2-TpU** ^**V**^ **PW**_**11**_). Further support for the formation of **2-TpU** ^**V**^ **PW**_**11**_ was obtained through electronic absorption spectroscopy (Figure 6). The UV–vis/NIR spectrum of the oxidized sample shows replacement of sharp f–f transitions characteristic of U^IV^ with broader f–f transitions at 1050, 1352, and 1586 nm that our team has observed previously in U^V^–POM complexes.^58,59^ There are also significant changes in the higher energy region of the spectrum. Notably, in the spectrum of **2-TpU** ^**V**^ **PW**_**11**_, a feature associated with U^IV^(5f) → W^VI^(5d) charge transfer is no longer obvious as intense absorption onsets at ca. 450 nm. These intense absorption features are credited to O­(2p) → U­(5f) LMCT, with the oxidation of the uranium center lowering the energy of these transitions, driving a red shift in this intense absorption feature when compared to **1-TpUPW**_**11**_. This clearly shows how the electronic interactions between the actinide and the POM change as the energy, size, and occupancy of the uranium 5f orbitals change.

**Figure 6:**
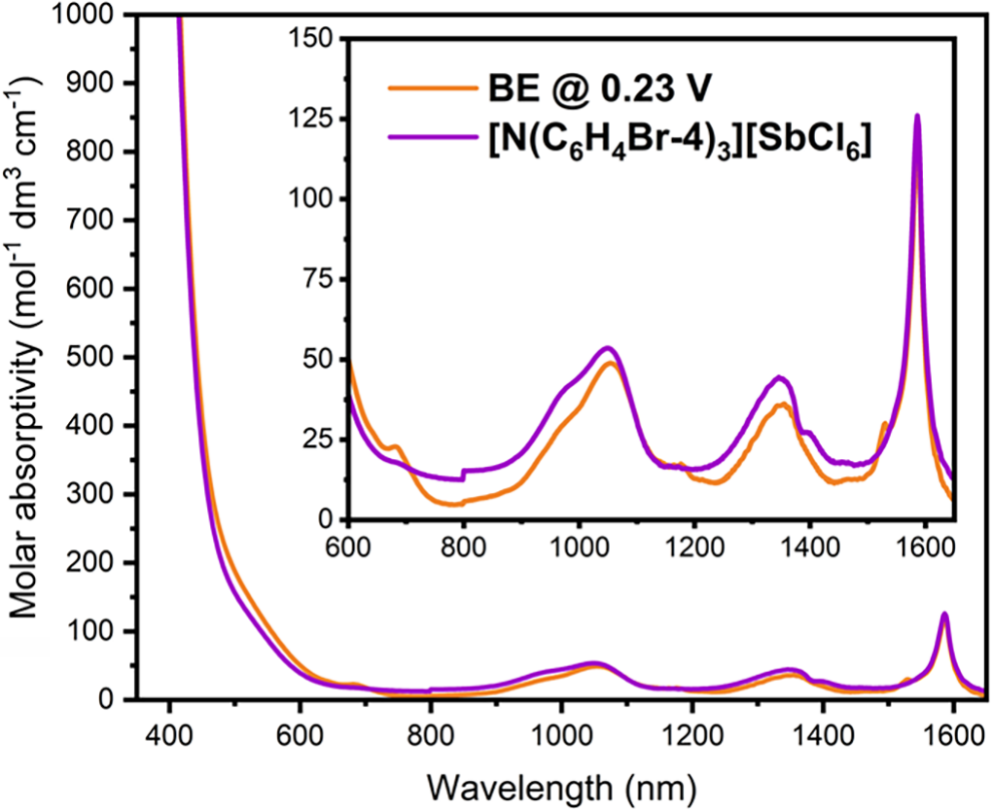
UV–vis-NIR spectra of MeCN solutions of **1-TpUPW**_**11**_ obtained after electrochemical (orange) or chemical (magenta) oxidation. Spectra were obtained at 21 °C.

To complement the electrochemical studies, the chemical oxidation of **1-TpUPW**_**11**_ was pursued. Treatment of a brown/red MeCN solution of **1-TpUPW**_**11**_ with one equivalent of [N­(C_6_H_4_Br-4)_3_]­[SbCl_6_] (*E*_Ox_ = 0.67 V vs Fc^+/0^ in MeCN) results in an immediate color change to the same yellow/orange color observed during CPE experiments. The solvent was removed, and the solid was washed with DCM and Et_2_O to remove the byproducts formed during the reaction. The product was further purified by crystallization (see the Experimental Section for more details). The UV–vis-NIR spectrum of the product was obtained (Figure 6, purple line). The spectrum is almost identical to that obtained from electrochemical experiments, confirming that chemical oxidation can also be used to access the U^V^ complex **2-TpU** ^**V**^ **PW**_**11**_.

Chemical oxidation provides access to **2-TpU** ^**V**^ **PW**_**11**_, which is not contaminated with an excess of electrolyte, enabling further characterization via ^1^H NMR and ^31^P NMR spectroscopy. As expected, the ^1^H NMR spectrum of **2-TpU** ^**V**^ **PW**_**11**_ (Figure S5) retains the same 8 resonances that were observed in the spectrum of **1-TpUPW**_**11**_, 4 peaks from the Tp^1–^ ligand and 4 peaks from the TBA^+^ cations. However, the signals assigned to the protons of the Tp^1–^ ligand are shifted toward their diamagnetic reference values (δ = 24.19, 6.94, 2,90, and −8.55 ppm). Similarly, the ^31^P NMR spectrum of **2-TpU** ^**V**^ **PW**_**11**_ shows a single peak at −80.71 ppm (Figure S6), which is shifted by 43.4 ppm compared to the corresponding peak in the ^31^P NMR spectrum of **1-TpUPW**_**11**_. Together, the NMR spectra show that the NMR active nuclei in asymmetric sandwich complexes are sensitive to changes in the oxidation state and electronic configuration of the actinide center (U^IV^, f^2^ vs U^V^, f^1^). Paramagnetic shifts in such systems arise from a combination of factors, including the distance between the NMR active nucleus and the U center, magnetic susceptibility anisotropy (Δχ), spin–orbit coupling, and metal–ligand interactions, all of which are perturbed upon oxidation.^96–98^ Further evidence of successful oxidation is given by infrared spectroscopy (Figure S7), where the shift in the positions of the ν­(PO) (1048 → 1058 cm^–1^) and ν­(W=O) (951 → 964 cm^–1^) to higher wavenumbers is consistent with a decrease in charge of the overall assembly.^99,100^

While single crystals of **2-TpU** ^**V**^ **PW**_**11**_ are readily obtained by vapor diffusion of Et_2_O into saturated solutions of the complex dissolved in MeCN, the high symmetry of the resultant crystal results in SCXRD diffraction experiments that fail to provide structural information. Unfortunately, attempts to obtain crystals that possess lower-symmetry space groups were unsuccessful.

After successfully isolating **2-TpU** ^**V**^ **PW**_**11**_, we attempted to further oxidize the actinide center to obtain the corresponding U^VI^ complex. CPE experiments performed at 1.18 V vs Fc^+/0^ with a fresh solution of **1-TpUPW**_**11**_ first led to a rapid color change from red/brown to orange, likely caused by the formation of **2-TpU** ^**V**^ **PW**_**11**_. The solution then gradually turns darker red/brown during the remainder of the experiment. We note that over the course of the CPE experiment, 2.24 × 10^–5^ moles of electrons were passed (Figure S8), corresponding to 2.24 e^–^ per molecule of **1-TpUPW**_**11**_. After CPE, the solution was analyzed by CV (Figure S9). Only small peaks consistent with the {TpUPW_11_O_39_} complex are observed, along with additional irreversible redox events, consistent with complex decomposition. Furthermore, the OCP of the resultant solution was measured to be only 0.08 V vs Fc^+/0^, inconsistent with the formation of [TpU^VI^PW_11_O_39_]^2–^. Electronic absorption spectroscopy of the solution following CPE gave limited insights into potential decomposition products, with only small peaks associated with **2-TpU** ^**V**^ **PW**_**11**_ and more intense absorption below 600 nm observed (Figure S10). Collectively, these results suggest that though [TpU^VI^PW_11_O_39_]^2–^ may be stable on the time scale of cyclic voltammetry experiments, it is insufficiently stable for isolation.

### Synthesis and Characterization of (TBA)_5_[TpUSiW_11_O_39_] (3-TpUSiW_11_)

Analytical data obtained for **1-TpUPW**_**11**_ successfully supported our hypothesis that increasing the overall anionic charge on a U-POM framework can be used to stabilize higher oxidation states of uranium. Unfortunately, the oxidation potential required to access the U^VI^ derivative was too high to afford isolation of this species. We hypothesized that further increases to the anionic charge on the framework could reduce the potential required to access the U^VI^ species, affording a potential pathway for isolation of the “non-uranyl” U^VI^−POM complex.

A convenient strategy for increasing the overall anionic charge of Keggin-type POM systems is through variation of the central heteroatom. In our case, swapping the lacunary [PW_11_O_39_]^7–^ fragment with the isostructural [SiW_11_O_39_]^8–^ fragment would result in an increase in anionic charge without significantly changing the local coordination environmental at the U center. Therefore, we next pursued the synthesis of (TBA)_5_[TpU^IV^SiW_11_O_39_] (**3-TpUSiW**_**11**_). To isolate the target compound, a significant modification of the synthetic procedure for the formation of the asymmetric sandwich-type complex is required (Scheme 2). First, (TBA)_4_H_4_[SiW_11_O_39_] was synthesized according to the methods of Balula and co-workers.^68^ Following workup, the protonated monolacunary POM was dissolved in MeCN along with TBA­(Tp).^69^ To this colorless solution, a yellow/brown solution of U^IV^[{N­(SiMe_3_)­(SiMe_2_CH_2_)}­{N­(SiMe_3_)_2_}_2_] dissolved in THF was added. The solution rapidly turns dark brown as the uranium precursor undergoes protonolysis, with the protons bound to the POM precursor reacting to give **3-TpUSiW**_**11**_ and hexamethyldisilazane as the only byproduct (Scheme 2). Following workup (see the Experimental Section for more details), a brown powder is obtained, which can be further crystallized from MeCN/DMF/Et_2_O.

**Scheme 2:**
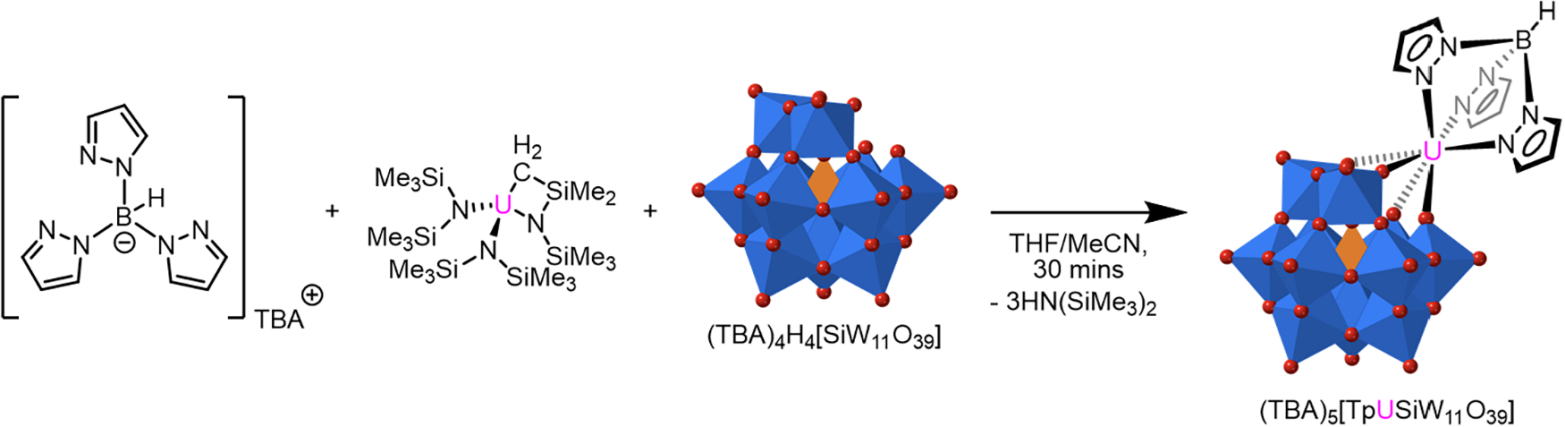
Synthetic Approach Used to Produce (TBA)_5_[TpUSiW_11_O_39_] (**3-TpUSiW**_**11**_)

The formation of **3-TpUSiW**_**11**_ was confirmed by ^1^H NMR spectroscopy. The obtained spectrum is shown in Figure 7 with a comparison to the ^1^H NMR spectrum of **1-TpUPW**_**11**_. It is immediately apparent that the two spectra are very similar, providing convincing evidence of the formation of **3-TpUSiW**_**11**_. Complex **3-TpUSiW**_**11**_ features four paramagnetically shifted and broadened resonances (in a 3:3:3:1 ratio, highlighted in red in Figure 7) assigned to the Tp ligand. The small discrepancies (ca. 0.5–1.5 ppm) in the exact chemical shifts of these peaks may reflect minor differences in the overall structure, with the chemical shifts of NMR-active nuclei being very sensitive to both distance and orientation relative to the paramagnetic metal center (U^IV^, f^*2*^) or the influence of the change in the overall charge on the assembly. The four additional resonances, observed at 0–4 ppm, are assigned to the 5 TBA cations that balance the charge of the anionic complex. Additional confirmation of the formation of **3-TpUSiW**_**11**_ was obtained via an elemental analysis. Unfortunately, although efforts to obtain single crystals were successful, they consistently possessed either hexagonal or cubic space groups, which prevented the extraction of useful structural information from SCXRD experiments.

**Figure 7:**
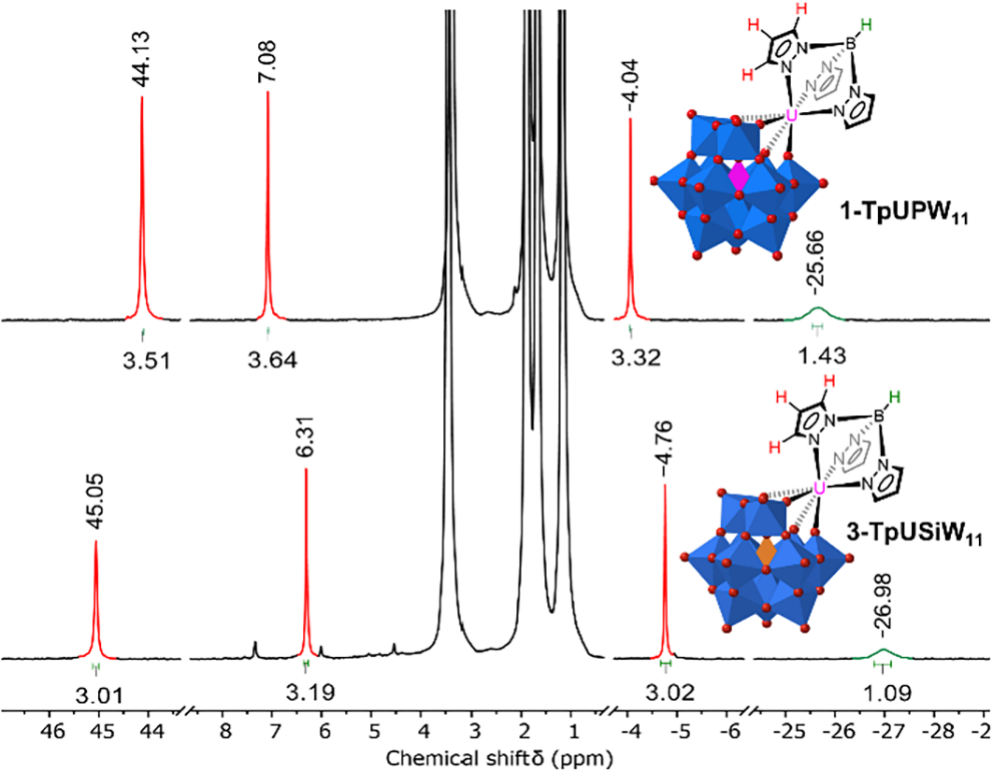
Obtained ^1^H NMR spectra of **1-TpUPW**_**11**_ (top) and **3-TpUSiW**_**11**_ (bottom), along with a pictorial representation of the structures. Spectra were obtained at room temperature in CD_3_CN.

Formation of **3-TpUSiW**_**11**_ was further confirmed by UV–vis/NIR and infrared spectroscopy. The UV–vis/NIR spectrum of **3-TpUSiW**_**11**_ (Figure S11) is extremely similar to that of **1-TpUPW**_**11**_, with f–f transitions associated with U^IV^ observed at 682 nm, 1092–1150 nm, and 1352–1460 nm (see the Experimental Section for exact wavelengths). The similarity of the position and shape of these transitions suggests that the coordination environment of the U^IV^ center is almost identical in both complexes. Furthermore, observation of a U^IV^(5f) → W^VI^(5d) charge transfer process at 484 nm (ε = 519 M^–1^ cm^–1^) provides further evidence of direct evidence of uranium binding to the POM. The infrared spectrum of **3-TpUSiW**_**11**_ (Figure S12) confirms the retention of the [SiW_11_O_39_]^8–^ structure upon reaction with uranium, with the 600–1600 cm^–1^ region of the spectrum closely matching previously reported spectra of [MSiW_11_O_39_]^n–^ complexes.^101,102^

To investigate the influence the change in overall anionic charge has on the redox properties of the assembly, the CV of **3-TpUSiW**_**11**_ was recorded. The CV is shown in Figure 8 along with the CV of **1-TpUPW**_**11**_ obtained under the same conditions (1 mM POM, 0.1 M TBA­(PF_6_) in MeCN at room temperature). The CV of **3-TpUSiW**_**11**_ contains four redox events, two reductions (*E*_1/2_ = −2.38, −1.90 V vs Fc^+/0^) and two oxidations (*E*_1/2_ = −0.48; 0.57 V vs Fc^+/0^). All of the events observed in the CV of **3-TpUSiW**_**11**_ are cathodically shifted (0.43–0.47 V) in comparison to that of **1-TpUPW**_**11**_, supporting our original hypothesis that increasing the anionic charge of the U-POM complex renders oxidation more energetically accessible. This occurs, however, at the cost of making the reduction of this complex more energetically demanding (W-based reduction events are also shifted by ca. 0.44 V). We note that this cathodic shift in the positions of the reduction events of **3-TpUSiW**_**11**_ prevents the observation of a third reduction event within the accessible electrochemical window, with this event presumably occurring at more negative potentials.

**Figure 8:**
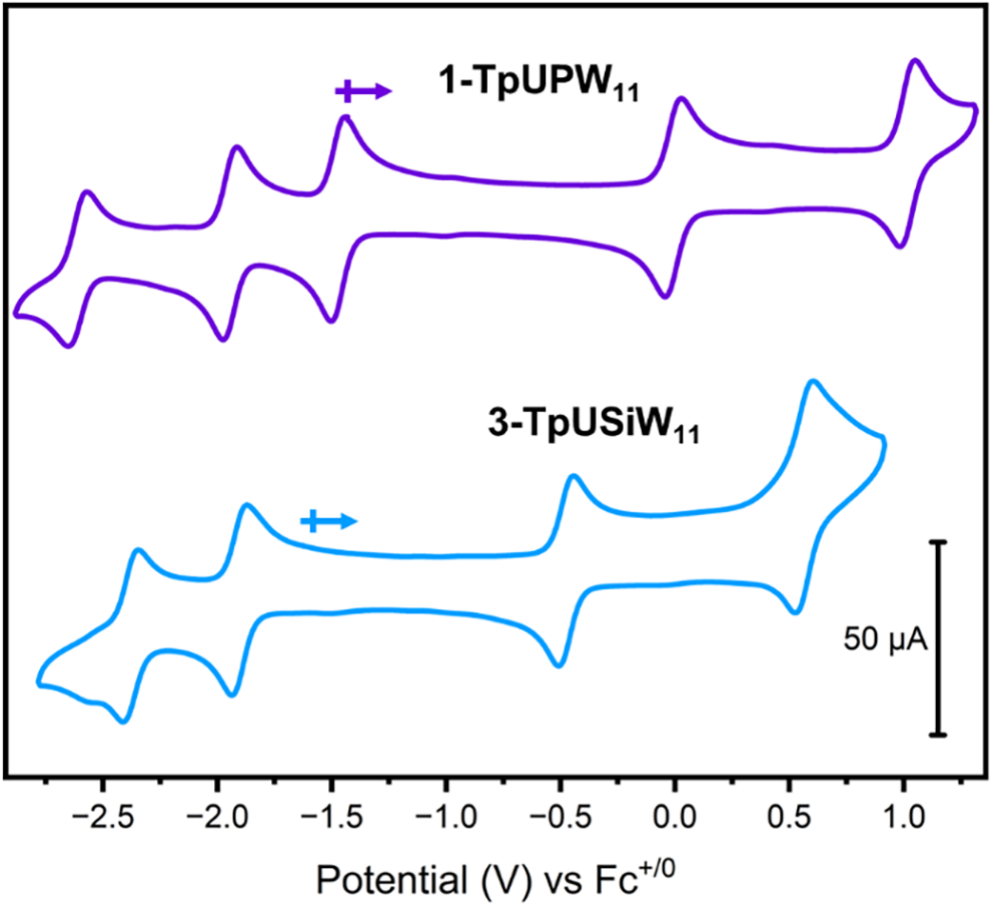
CVs of **1-TpUPW**_**11**_ (purple) and **3-TpUSiW**_**11**_ (blue). CVs were obtained in a solution of 0.1 M TBA­(PF_6_) in MeCN at 200 mv/s. The analyte concentration was 1 mM.

### Investigating the Oxidation of 3-TpUSiW_11_

To explore whether the change in the positions of the uranium-based redox events translates to increases in stability of the U^V^ and, in particular, the U^VI^-containing analogues of **3-TpUSiW**_**11**_, both chemical and electrochemical oxidation of the assembly were performed.

Electrochemical oxidation of **3-TpUSiW**_**11**_ targeting the U^V^ analogue was first investigated. CPE of a solution of the complex in MeCN (1 mM **3-TpUSiW**_**11**_, 0.1 M TBA­(PF_6_)) at −0.15 V vs Fc^+/0^ led to a color change from brown to yellow/orange. During the experiment, ca. 1.1 × 10^–5^ moles of electrons (1.1 e^–^ per molecule of **3-TpUSiW**_**11**_) were passed, consistent with one electron oxidation of **3-TpUSiW**_**11**_ to (TBA)_4_[TpU^V^SiW_11_O_39_] (**4-TpU** ^**V**^ **SiW**_**11**_) (Figure S13). Formation of the desired U^V^ complex was confirmed by cyclic voltammetry and UV–vis-NIR spectroscopy. The CV of the resulting solution (Figure S14) appeared almost identical to that of pristine **3-TpUSiW**_**11**_, with the main difference being a shift in the OCP from −1.12 V vs Fc^+/0^ prior to electrolysis to −0.37 V vs Fc^+/0^ after electrolysis. This shift of the OCP to a more positive position than the U^V/IV^ redox couple (−0.48 vs Fc^+/0^) supports the formation of **4-TpU** ^**V**^ **SiW**_**11**_. Further analysis of the resulting solution by UV–vis-NIR spectroscopy (Figure 9, orange) shows that the characteristic sharp f–f transitions associated with the U^IV^ center of **3-TpUSiW**_**11**_ (Figure S15) are replaced with broader transitions at 920, 1048, and 1342 nm, and a sharp transition at 1564 nm. Similar peaks are observed in previously reported U^V^ complexes^58,59^ and **2-TpU** ^**V**^ **PW**_**11**_ discussed above, supporting successful oxidation of **3-TpUSiW**_**11**_ to **4-TpU** ^**V**^ **SiW**_**11**_. We note that all three absorptions of **4-TpU** ^**V**^ **SiW**_**11**_ are blue-shifted in comparison to that of **2-TpU** ^**V**^ **PW**_**11**_ (Figure S16). We postulate that this is caused by the change in the strength of the interactions between the U^V^ center and the POM anion as the charge is varied. The more highly anionic [SiW_11_O_39_]^8–^ is likely to form stronger bonding interactions with the U^V^ center, which stabilize the partially filled 5f orbital. This in turn will increase the energy difference between the filled and empty 5f orbitals, driving the observed blue shift in transitions involved with moving between these orbitals.

**Figure 9:**
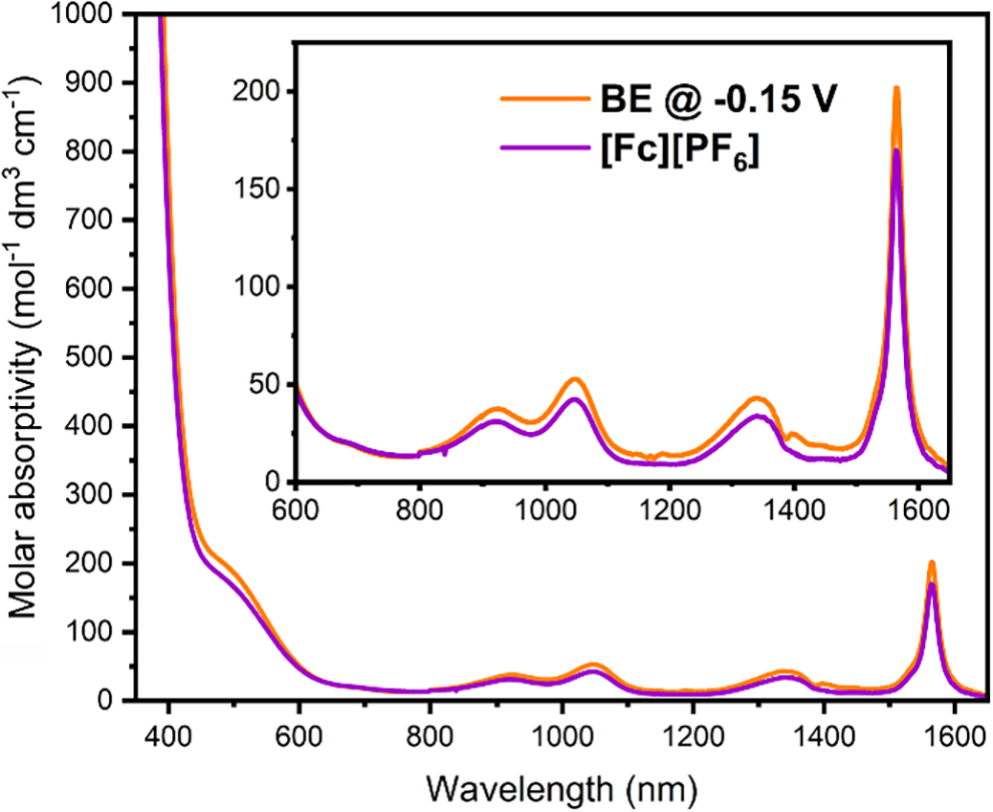
UV–vis-NIR spectra of MeCN solutions of **3-TpUSiW**_**11**_ obtained after electrochemical (orange) or chemical (magenta) oxidation. Spectra were obtained at 21 °C.

Encouraged by the results of electrochemical oxidation of **3-TpUSiW**_**11**_ to **4-TpU** ^**V**^ **SiW**_**11**_, we moved on to chemical oxidation experiments. To achieve this, **3-TpUSiW**_**11**_ was treated with one equivalent of [Fc]­[PF_6_] in MeCN. This led to an instant color change from brown to orange as **3-TpUSiW**_**11**_ was oxidized to **4-TpU** ^**V**^ **SiW**_**11**_, and [Fc]^+^ was consumed. The solvent was removed under reduced pressure to leave a tacky solid. The solid was triturated with Et_2_O and DCM to remove Fc and TBA­(PF_6_) byproducts, before crystallization from MeCN/DMF/Et_2_O (see the Experimental Section for more details). The product was first characterized by UV–vis-NIR spectroscopy (Figure 9, magenta) and cyclic voltammetry (Figure S16). The obtained spectrum and voltammogram are almost identical to those obtained after electrochemical one-electron oxidation, consistent with the successful formation of the same U^V^ complex (i.e., **4-TpU** ^**V**^ **SiW**_**11**_). Complex **4-TpU** ^**V**^ **SiW**_**11**_ was further characterized by ^1^H NMR spectroscopy (Figure S17), showing the characteristic spectrum of a Tp-U-POM complex; four paramagnetically shifted resonances are observed in a 3:3:3:1 ratio (δ = −4.03, 4.56, 6.29, and 18.57 ppm), assigned to the Tp ligand, and four peaks at 0–4 ppm assigned to the TBA^+^ cations. Interestingly, there are significant differences between the positions of the resonances assigned to the Tp ligand in the spectra of **2-TpU** ^**V**^ **PW**_**11**_ and **4-TpU** ^**V**^ **SiW**_**11**_, with the peaks in the ^1^H NMR spectrum of **4-TpU** ^**V**^ **SiW**_**11**_ moving closer to the expected diamagnetic values. Given shifting caused by the presence of paramagnetic metal centers (in this case U^V^, f^1^) is extremely sensitive to the distance of NMR active nuclei to the paramagnetic metal center, a decrease in this displacement is consistent with an increase in the distance between the protons of the Tp ligand and U^V^ center in **4-TpU** ^**V**^ **SiW**_**11**_ when compared to **2-TpU** ^**V**^ **PW**_**11**_. We hypothesize that the observed shift of signals is a result of the presence of stronger interactions between the more highly anionic [SiW_11_O_39_]^8–^ unit and the U^V^ ion compared to the corresponding interactions between U^V^ and [PW_11_O_39_]^7–^.

Attention was then turned to the generation of the U^VI^-containing complex (TBA)_3_[TpU^VI^SiW_11_O_39_]. To investigate electrochemical oxidation, a potential of 0.75 V vs Fc^+/0^ was applied to a solution of **4-TpU^V^SiW**_**11**_, causing the solution to become more intensely orange. Integration of the plot of current vs time (Figure S18) showed that ca. 1.3 × 10^–5^ moles of electrons (1.3 e^–^ per molecule of **4-TpU** ^**V**^ **SiW**_**11**_) were passed during the experiment, consistent with further oxidation of **4-TpU** ^**V**^ **SiW**_**11**_ by one electron to (TBA)_3_[TpU^VI^SiW_11_O_39_]. The CV of the resulting solution (Figure S19) was very similar to that obtained prior to electrolysis; the OCP shifts from −0.34 V vs Fc^+/0^ to 0.58 V vs Fc^+/0^, now appearing to the right of the U^VI/V^ redox couple, consistent with the formation of (TBA)_3_[TpU^VI^SiW_11_O_39_]. Some minor differences in the reversibility of the redox events, particularly the second reduction event (observed at ca. −2.4 V vs Fc^+/0^), are observed. This suggests that (TBA)_3_[TpU^VI^SiW_11_O_39_] is not completely stable when cycling between the four redox states accessible to the cluster on the CV time scale and may be a sign of the limited solution stability of (TBA)_3_[TpU^VI^SiW_11_O_39_] when compared to the U^IV^ or U^V^ containing analogues. UV–vis-NIR spectroscopy was performed on the solution and is shown in Figure 10. The lower energy region (800–1650 nm) shows only small peaks at 920, 1048, 1342, and 1564 nm caused by a small amount of residual **4-TpU** ^**V**^ **SiW**_**11**_. The absence of any new peaks in this region is consistent with the formation of a U^VI^ species, which has empty 5f orbitals and thus f–f transitions are not possible. Interestingly, there is a new intense absorption that onsets at approximately 600 nm. It is postulated that this is caused by the shifting of the O­(2p) → U­(5f) LMCT process as the uranium center is oxidized. Collective analysis of the UV–vis/NIR spectra of **3-TpUSiW**_**11**_ (Figure S11), **4-TpU** ^**V**^ **SiW**_**11**_ (Figure 9), and the spectrum obtained post electrolysis at 0.75 V vs Fc^+/0^ (Figure 10) shows that the onset of the intense absorption feature moves from <400 nm to ca. 600 as uranium is oxidized from +4 to +6. This is consistent with the idea that oxidation of uranium, and concomitant removal of the valence f-electrons, lowers the energy of the valence 5f orbitals, and in turn lowers the energy of O­(2p) → U­(5f) LMCT. This clearly illustrates how changes in the actinide valency drive changes in metal–ligand interactions.

**Figure 10:**
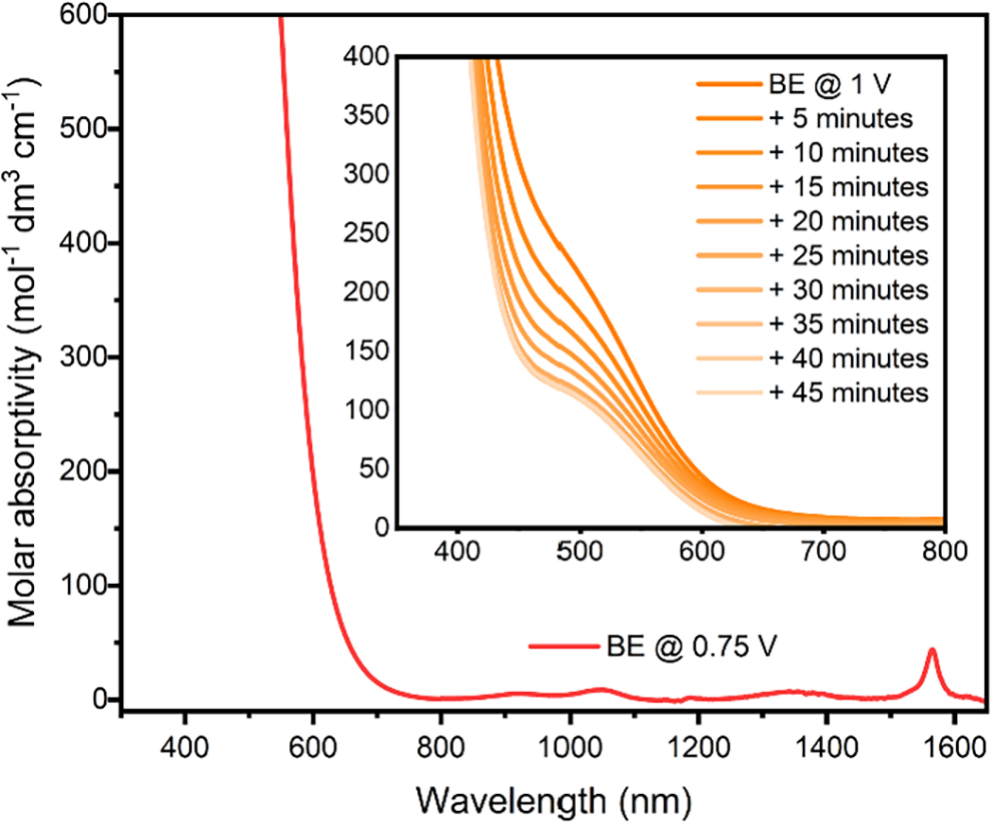
UV–vis-NIR spectra of MeCN solutions of **4-TpU** ^**V**^ **SiW**_**11**_ obtained after electrochemical oxidation at 0.75 V vs Fc^0/+^ (main plot) and 1 V vs Fc^0/+^ (inset). Spectra were obtained at 21 °C.

During the characterization of the purported U^VI^ POM complex, it was noted that the color of the solutions obtained from CPE faded over time. We therefore investigated the solution stability of (TBA)_3_[TpU^VI^SiW_11_O_39_]. To do this, the electrochemical oxidation of **4-TpU** ^**V**^ **SiW**_**11**_ was repeated using a more positive potential (1 V vs Fc^+/0^) to minimize the time required to generate a substantial quantity of (TBA)_3_[TpU^VI^SiW_11_O_39_]. After performing CPE for ∼10 min, the solution was transferred to a cuvette and the UV–vis-NIR spectrum was monitored as a function of time (Figure 10, inset). The shorter duration of the electrolysis leads to a mixture of **4-TpU** ^**V**^ **SiW**_**11**_ and (TBA)_3_[TpU^VI^SiW_11_O_39_], as evidenced by the presence of both the f–f transitions of **4-TpU** ^**V**^ **SiW**_**11**_ and the new charge-transfer feature discussed above (full spectral range shown in Figure S20). While monitoring the UV–vis-NIR spectrum, the LMCT process that onsets around 600 nm recedes and is no longer visible after 45 min. At this point, only peaks assigned to **4-TpU** ^**V**^ **SiW**_**11**_ remain. This suggests that electrochemically generated (TBA)_3_[TpU^VI^SiW_11_O_39_] decomposes in solution at room temperature, while **4-TpU** ^**V**^ **SiW**_**11**_ is solution-stable. Notably, the features associated with **4-TpU** ^**V**^ **SiW**_**11**_ do not grow as the LMCT associated with (TBA)_3_[TpU^VI^SiW_11_O_39_] recedes, indicating that (TBA)_3_[TpU^VI^SiW_11_O_39_] does not reform **4-TpU** ^**V**^ **SiW**_**11**_ as it decomposes.

In an attempt to stabilize (TBA)_3_[TpU^VI^SiW_11_O_39_], alternative solvents for electrochemical studies were explored. Unfortunately, the limited solubility of **4-TpU** ^**V**^ **SiW**_**11**_ limits options to highly polar solvents, and therefore, we investigated the use of DMF. Interestingly, the CV of **4-TpU** ^**V**^ **SiW**_**11**_ in DMF (Figure S21) shows a cathodic shift in the positions of the U^V/IV^ (−0.67 V vs Fc^+/0^) and U^VI/V^ (0.43 V vs Fc^+/0^). Performing similar bulk electrolysis experiments in DMF, however, only provides more evidence of (TBA)_3_[TpU^VI^SiW_11_O_39_] instability. CPE of a solution of **4-TpU** ^**V**^ **SiW**_**11**_ in DMF at 0.69 V vs Fc^+/0^ (Figure S22) initially leads to the formation of an intensely orange solution. Analysis of the solution post CPE by CV shows evidence of decomposition (Figure S23), while the OCP shifts cathodically from 0.46 V and the color of the solution fades over time, suggesting that the stability of (TBA)_3_[TpU^VI^SiW_11_O_39_] is even lower in DMF than in MeCN.

Given the results from electrochemical oxidation reactions, which indicated that (TBA)_3_[TpU^VI^SiW_11_O_39_] can be formed, we also attempted chemical oxidation reactions. First, we treated **4-TpU** ^**V**^ **SiW**_**11**_ with one equivalent of [N­(C_6_H_4_Br-4)_3_]­[SbCl_6_] in MeCN. While stirring for ca. 15 min at room temperature, the blue color of [N­(C_6_H_4_Br-4)_3_]­[SbCl_6_] fades and a dark orange solution is obtained, supporting consumption of the oxidizing agent and formation of (TBA)_3_[TpU^VI^SiW_11_O_39_]. After workup (see the Experimental Section), ^1^H NMR reveals the presence of three peaks in a 1:1:1 ratio at 6.76, 8.31, and 8.53 ppm (Figure S24). These peaks are in the appropriate position to be assigned to the aromatic protons of the Tp ligand of (TBA)_3_[TpU^VI^SiW_11_O_39_], which is now diamagnetic (U^VI^, *f* ^0^). However, these peaks are accompanied by peaks previously assigned to **4-TpU** ^**V**^ **SiW**_**11**_, suggesting the reaction does not go to completion. Performing the reaction with two equivalents of [N­(C_6_H_4_Br-4)_3_]­[SbCl_6_] does lead to complete consumption of **4-TpU** ^**V**^ **SiW**_**11**_; however, there is no significant increase in the size of the peaks at 6.76, 8.31, and 8.53 ppm (Figure S25). Furthermore, integration of these peaks with respect to the peaks associated with the TBA cations (1–3.2 ppm) reveals that the peaks tentatively assigned to the Tp ligand of (TBA)_3_[TpU^VI^SiW_11_O_39_] are far too small to suggest clean formation of (TBA)_3_[TpU^VI^SiW_11_O_39_], even when accounting for the presence of the byproduct TBA­[SbCl_6_]. Therefore, though these ^1^H NMR spectra support the ability of [N­(C_6_H_4_Br-4)_3_]­[SbCl_6_] to oxidize **4-TpU** ^**V**^ **SiW**_**11**_ and the formation of (TBA)_3_[TpU^VI^SiW_11_O_39_], it appears that (TBA)_3_[TpU^VI^SiW_11_O_39_] is not the major species in solution even after short periods in solution. Carrying out reactions between **4-TpU** ^**V**^ **SiW**_**11**_ and [N­(C_6_H_4_Br-4)_3_]­[SbCl_6_] at low temperature, either by mixing at −30 °C and allowing the solution to warm to room temperature while monitoring by ^1^H NMR spectroscopy (Figure S26) or maintaining −40 °C throughout the reaction and monitoring by UV–vis-NIR spectroscopy (Figure S27), showed no improvements, with [N­(C_6_H_4_Br-4)_3_]­[SbCl_6_] consumed without total consumption of **4-TpU** ^**V**^ **SiW**_**11**_ or formation of significant quantities of (TBA)_3_[TpU^VI^SiW_11_O_39_].

## Conclusions

In summary, a series of asymmetric hybrid organic–inorganic uranium sandwich-type complexes have been synthesized and characterized. Asymmetric sandwich-type complexes are rare in the literature, with only a couple of examples known,^26,29^ likely due to difficulties in controlling solution speciation and product distribution. Utilization of lacunary Keggin clusters in combination with an organic Tp ligand provides access to more highly anionic systems than our previously reported (TBA)_2_[U^IV^{M_4_O_13_(OMe)_4_MoNO}_2_] (M = Mo or W) systems. These new systems appear to be better at supporting high valent uranium, with U^V/IV^ redox couples shifted cathodically by ca. 0.75–1.25 V vs Fc^+/0^, while highly stable U^V^ complexes (i.e., **2-TpU** ^**V**^ **PW**_**11**_ and **4-TpU** ^**V**^ **SiW**_**11**_) are readily isolated and studied. The redox properties of the complexes appear to be highly charge-dependent, clearly illustrating how charge can be used as a design criterion to modulate both actinide- and framework-based redox chemistry. Though U^VI/V^ redox couples are also visible in the CVs of these asymmetric sandwich-type complexes, attempts to isolate the U^VI^-containing derivatives were ultimately unsuccessful. However, the increase in stability of (TBA)_3_[TpU^VI^SiW_11_O_39_] compared to (TBA)_2_[TpU^VI^PW_11_O_39_] suggests that further increases in anionic charge could provide a pathway to the isolation of a stable U-POM complex containing “non-uranyl” U^VI^. This is the subject of ongoing work, with alternative synthetic strategies being explored to access more highly anionic assemblies.

## Supplementary Material



## Notes

Crystallographic details, additional crystal structure descriptions, ^1^H and ^31^P NMR spectra of all prepared compounds, electronic absorption spectra, and electrochemical data (PDF)
