# Supplementary material for: Tuning Uranium Redox Chemistry in Asymmetric Polyoxometalate Complexes: Access to U(IV), U(V), and Transient U(VI)
Source: Inorg Chem. 2026 Jun 11;65(25):14169–82. doi: 10.1021/acs.inorgchem.6c01673 (PMC13321305; doi:10.1021/acs.inorgchem.6c01673)
Supplement: Supplementary file 1 [file ic6c01673_si_001.pdf]

Electronic Supporting Information

Tuning Uranium Redox Chemistry in Asymmetric Polyoxometalate Complexes: Access to U(IV), U(V), and Transient U(VI)

Dominic Shiels, William W. Brennessel, and Ellen M. Matson*

Department of Chemistry, University of Rochester, Rochester NY 14627 USA

Corresponding Author Contact Information:

Ellen M. Matson: matson@chem.rochester.edu

Contents

Table S1. Continuous shape measure (CShM) analysis for **1-TpUPW$_{11}$** using SHAPE. Positions in Cartesian coordinates (x,y,z). S4

Table S2. Crystal data and structure refinement for **1-TpUPW$_{11}$**. S5

Figure S1. Infrared spectrum of **1-TpUPW$_{11}$**. S6

Figure S2. UV-Vis/NIR spectrum of $(TBA)_6[NaPW_{11}O_{39}]$ (1 mM) in MeCN. The spectrum was recorded at 21 °C. S6

Figure S3. Bulk electrolysis of a solution of **1-TpUPW$_{11}$** (1 mM) in MeCN (100 mM TBA(PF_6)) at 0.23 V vs $Fc^{+/0}$. S7

Figure S4. Post bulk electrolysis (0.23 V vs $Fc^{+/0}$) CV of **1-TpUPW$_{11}$** (1 mM) in MeCN (100 mM TBA(PF_6)). S7

Figure S5. ^{1}H NMR (500 MHz) of **2-TpU^{V}PW$_{11}$** in CD_3CN. Spectrum acquired at 21 °C. S8

Figure S6. ^{31}P NMR (202.4 MHz) of **2-TpU^{V}PW$_{11}$** in CD_3CN. Spectrum acquired at 21 °C. S8

Figure S7. Infrared spectrum of **2-TpU^{V}PW$_{11}$**. S9

Figure S8. Bulk electrolysis of a solution of **1-TpUPW$_{11}$** (1 mM) in MeCN (100 mM TBA(PF_6)) at 1.18 V vs $Fc^{+/0}$. S9

Figure S9. Post bulk electrolysis (1.18 V vs $Fc^{+/0}$) CV of **1-TpUPW$_{11}$** (1 mM) in MeCN (100 mM TBA(PF_6)). S10

Figure S10. Post bulk electrolysis (1.18 V vs $Fc^{+/0}$) UV-Vis/NIR spectrum of **1-TpUPW$_{11}$** (1 mM) in MeCN (100 mM TBA(PF_6)). Spectrum was recorded at 21 °C. S10

Figure S11. UV-Vis/NIR spectrum of **3-TpUSiW$_{11}$** (1 mM) in MeCN. Spectrum was recorded at 21 °C. S11

Figure S12. Infrared spectrum of **3-TpUSiW$_{11}$**. S11

Figure S13. Bulk electrolysis of a solution of **3-TpUSiW$_{11}$** (1 mM) in MeCN (100 mM TBA(PF_6)) at −0.15 V vs $Fc^{+/0}$. S12

Figure S14. Post bulk electrolysis (−0.15 V vs $Fc^{+/0}$) CV of **3-TpUSiW$_{11}$** (1 mM) in MeCN (100 mM TBA(PF_6)). S12

Figure S15. Comparison of the UV-Vis/NIR spectra of **2-TpU^{V}PW$_{11}$** and **4-TpU^{V}SiW$_{11}$** (both at 1 mM) in MeCN. Spectra were recorded at 21 °C. S13

Figure S16. CV of **4-TpU^{V}SiW$_{11}$** (1 mM) in MeCN (100 mM TBA(PF_6)). S13

Figure S17. ^{1}H NMR (500 MHz) of **4-TpU^{V}SiW$_{11}$** in CD_3CN. Spectrum acquired at 21 °C. S14

Figure S18. Bulk electrolysis of a solution of **4-TpU^{V}SiW$_{11}$** (1 mM) in MeCN (100 mM TBA(PF_6)) at 0.75 V vs $Fc^{+/0}$. S14

Figure S19. Post bulk electrolysis (0.75 V vs $Fc^{+/0}$) CV of **4-TpU^{V}SiW$_{11}$** (1 mM) in MeCN (100 mM TBA(PF_6)). S15

Figure S20. UV-Vis/NIR spectra of a solution of **4-TpU^{V}SiW$_{11}$** (1 mM) in MeCN over one hour at 21 °C (room temperature). S15

Figure S21. CV of **4-TpU^{V}SiW$_{11}$** (1 mM) in DMF (100 mM TBA(PF_6)). S16

Figure S22. Bulk electrolysis of a solution of **4-TpU^{V}SiW$_{11}$** (1 mM) in DMF (100 mM TBA(PF_6)) at 0.69 V vs $Fc^{+/0}$. S16

Figure S23. Post bulk electrolysis (0.69 V vs $Fc^{+/0}$) CV of **4-TpU^{V}SiW$_{11}$** (1 mM) in DMF (100 mM TBA(PF_6)). S17

Figure S24. ^{1}H NMR (500 MHz) obtained after reaction of **4-TpU^{V}SiW$_{11}$** with 1 eq. of [N(C_6H_4Br-4)$_3$][$SbCl_6$] and work-up (see experimental section for details). The spectrum was acquired in CD_3CN at 21 °C. The peak at 6.30 ppm assigned to unreacted **4-TpU^{V}SiW$_{11}$**. The peaks at 5.45 ppm and ca. 1.1 ppm/3.4 ppm are assigned to DCM and Et_2O. S17

Figure S25. ^{1}H NMR (500 MHz) obtained after reaction of **4-TpU^{V}SiW$_{11}$** with 2 eq. of [N(C_6H_4Br-4)$_3$][$SbCl_6$] and work-up (see experimental section for details). The spectrum was acquired in CD_3CNat 21 °C. Peaks at ca. 1.1 and 3.4 ppm are assigned to Et_2O. S18

Figure S26. ^{1}H NMR (500 MHz) spectra following the reaction of **4-TpU^{V}SiW$_{11}$** with 1 eq. of [N(C_6H_4Br-4)$_3$][$SbCl_6$]. A solution of [N(C_6H_4Br-4)$_3$][$SbCl_6$] in CD_3CN was cooled to −30 °C and then treated with a solution of **4-TpU^{V}SiW$_{11}$** at −30 °C. The solution was allowed to warm to room temperature as ^{1}H NMR spectra were recorded. S18

Figure S27. UV-Vis/NIR spectra following the reaction of **4-TpU^{V}SiW$_{11}$** with 1 eq. of [N(C_6H_4Br-4)$_3$][$SbCl_6$]. A solution of [N(C_6H_4Br-4)$_3$][$SbCl_6$] in MeCN was cooled to −40 °C and then treated with a solution of **4-TpU^{V}SiW$_{11}$** at −40 °C. The solution was held at −40 °C and stirred as spectra were recorded. Features associated with **4-TpU^{V}SiW$_{11}$** (i.e. f-f transitions at 1048 nm, 1340 nm, and 1564 nm) only grow after initial mixing (T = 0) showing incomplete consumption of the starting material. Also charge transfer features at <600 nm only recedes during the experiment, suggesting if any (TBA)$_3$[TpU^{VI}SiW$_{11}$O$_{39}$] forms, it decomposes during the reaction. S19

Table S1. Continuous shape measure (CShM) analysis for **1-TpUPW$_{11}$** using SHAPE. Positions in Cartesian coordinates (x,y,z).

Structure	**1-TpUPW$_{11}$**		
U	7.9851	29.9114	12.1828
O	9.9666	29.9402	11.1560
O	7.4526	28.4912	10.5627
O	5.9643	30.7854	11.9145
O	8.3519	32.0734	12.4853
N	9.3803	27.8416	12.9375
N	6.4392	28.3887	13.5248
N	8.5406	30.1389	14.6649

COC-7 (Capped octahedron) Ideal structure CShM = 0.64977

U	M	8.0337	29.8212	12.4359
O	L2	9.8482	30.0671	10.9668
O	L5	7.4381	28.3906	10.6725
O	L3	6.0034	30.7504	11.7110
O	L1	8.4690	32.1241	12.5708
N	L6	9.3090	27.9062	12.9021
N	L7	6.4553	28.4133	13.4545
N	L4	8.5240	30.0978	14.7150

CTPR-7 (Capped trigonal prism) Ideal structure CShM = 1.69375

U	M	7.9570	29.7906	12.4094
O	L2	10.0557	30.1307	11.4459
O	L3	7.4576	28.4831	10.5413
O	L7	5.9019	30.6251	11.6822
O	L6	8.2375	32.1063	12.4954
N	L1	9.0850	27.7881	12.8170
N	L5	6.3939	28.4996	13.5664
N	L4	8.9919	30.1472	14.4710

Table S2. Crystal data and structure refinement for **1-TpUPW$_{11}$**.

Empirical formula	$C_{80}H_{171}BN_{11}O_{41}PUW_{11}$	
Formula weight	4245.43	
Temperature	100.00(10) K	
Wavelength	1.54184 Å	
Crystal system	monoclinic	
Space group	$P2_1/n$	
Unit cell dimensions	*a* = 14.99900(10) Å	a = 90°
	b = 51.4755(3) Å	b = 104.0340(10)°
	c = 15.48300(10) Å	g = 90°
Volume	11597.32(14) Å^3	
Z	4	
Density (calculated)	2.431 Mg/m^3	
Absorption coefficient	24.209 mm^{-1}	
F(000)	7928	
Crystal color, morphology	orange-brown, plate	
Crystal size	0.093 x 0.085 x 0.022 mm^3	
Theta range for data collection	3.065 to 80.525°	
Index ranges	$-19 \le h \le 19$, $-65 \le k \le 65$, $-19 \le l \le 14$	
Reflections collected	197916	
Independent reflections	24995 [*R*(int) = 0.0789]	
Observed reflections	22647	
Completeness to theta = 74.504°	99.9%	
Absorption correction	Multi-scan	
Max. and min. transmission	1.00000 and 0.21137	
Refinement method	Full-matrix least-squares on F^2	
Data / restraints / parameters	24995 / 433 / 1522	
Goodness-of-fit on F^2	1.060	
Final *R* indices [*I*>2sigma(*I*)]	*R*1 = 0.0579, *wR*2 = 0.1345	
R indices (all data)	*R*1 = 0.0626, *wR*2 = 0.1370	
Largest diff. peak and hole	3.627 and -2.752 e.Å^{-3}	

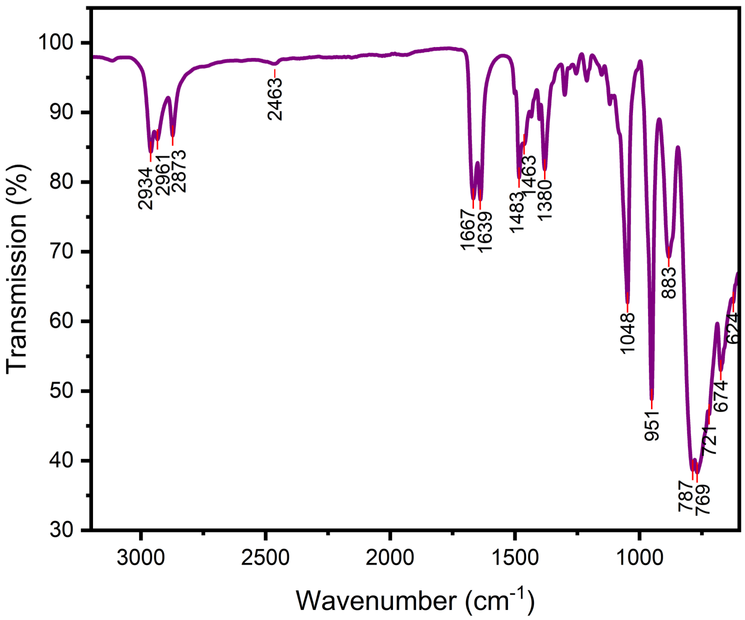

Figure S1. Infrared spectrum of **1-TpUPW$_{11}$**.

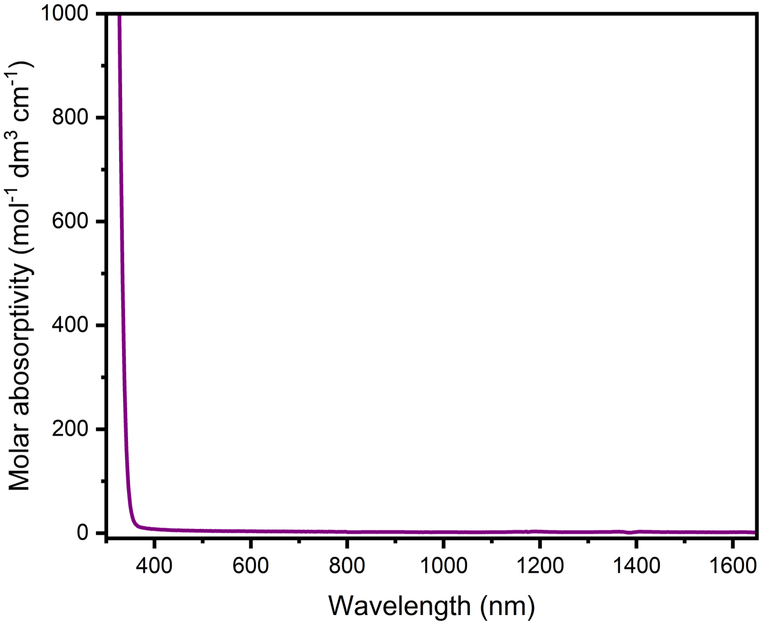

Figure S2. UV-Vis/NIR spectrum of $(TBA)_6[NaPW_{11}O_{39}]$ (1 mM) in MeCN. The spectrum was recorded at 21 °C.

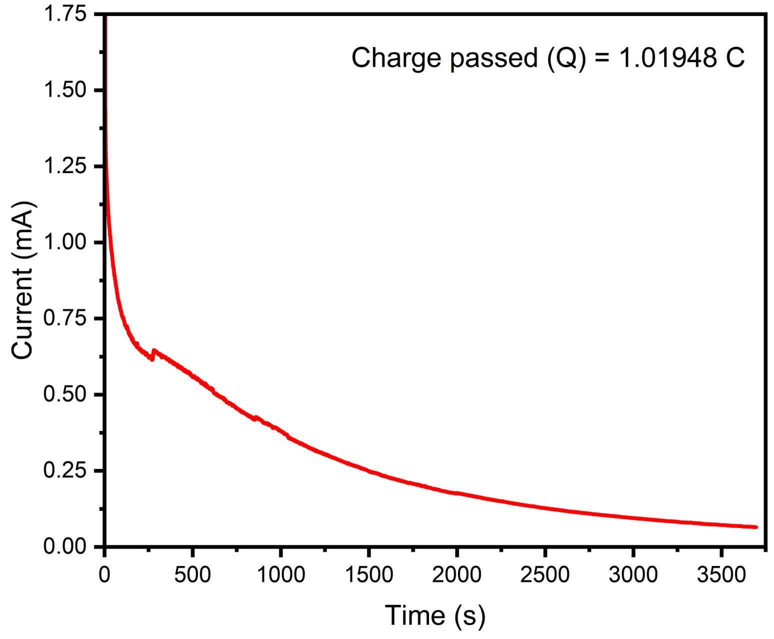

Figure S3. Bulk electrolysis of a solution of **1-TpUPW$_{11}$** (1 mM) in MeCN (100 mM TBA(PF_6)) at 0.23 V vs $Fc^{+/0}$.

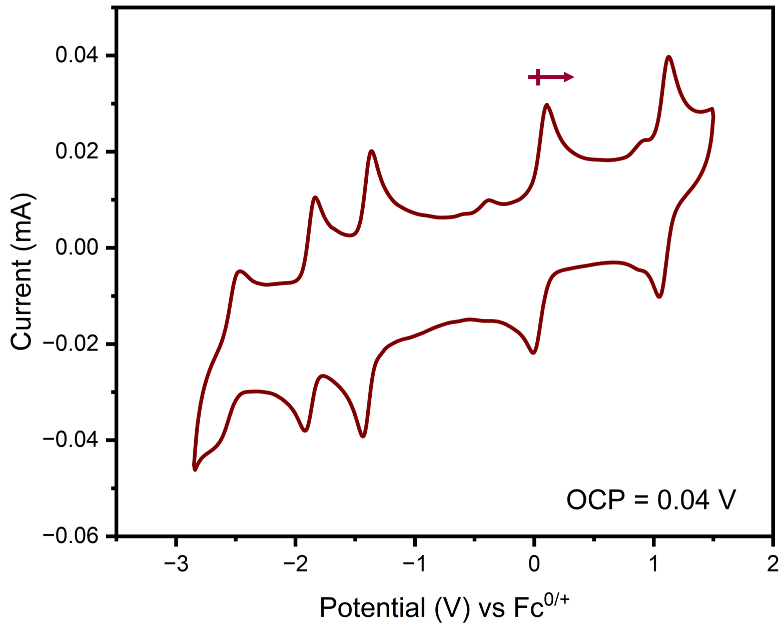

Figure S4. Post bulk electrolysis (0.23 V vs $Fc^{+/0}$) CV of **1-TpUPW$_{11}$** (1 mM) in MeCN (100 mM TBA(PF_6)).

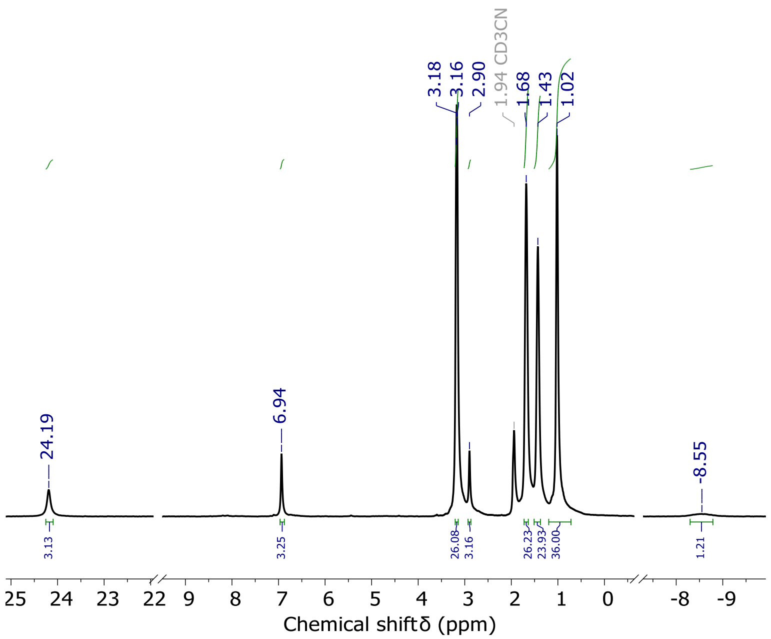

Figure S5. ^{1}H NMR (500 MHz) of **2-TpU^{V}PW$_{11}$** in CD_3CN. Spectrum acquired at 21 °C.

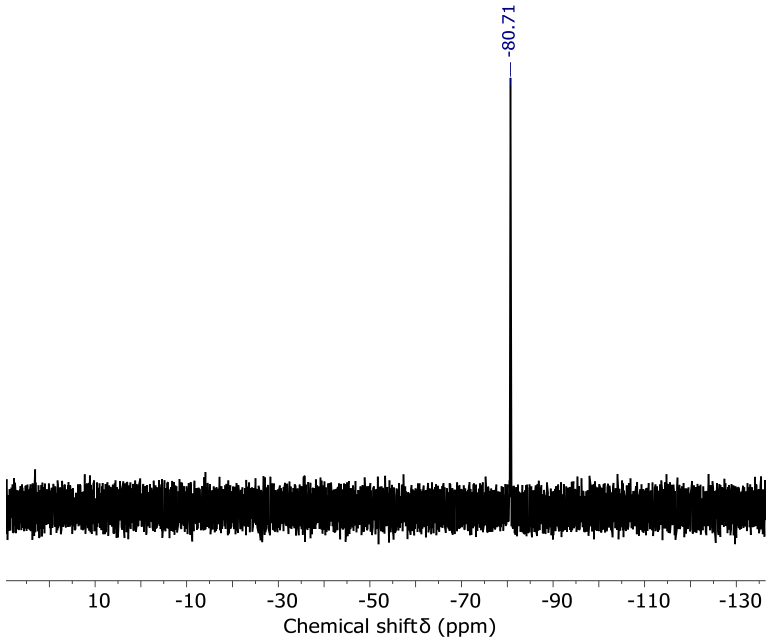

Figure 6. ^{31}P NMR (202.4 MHz) of **2-TpU^{V}PW$_{11}$** in CD_3CN. Spectrum acquired at 21 °C.

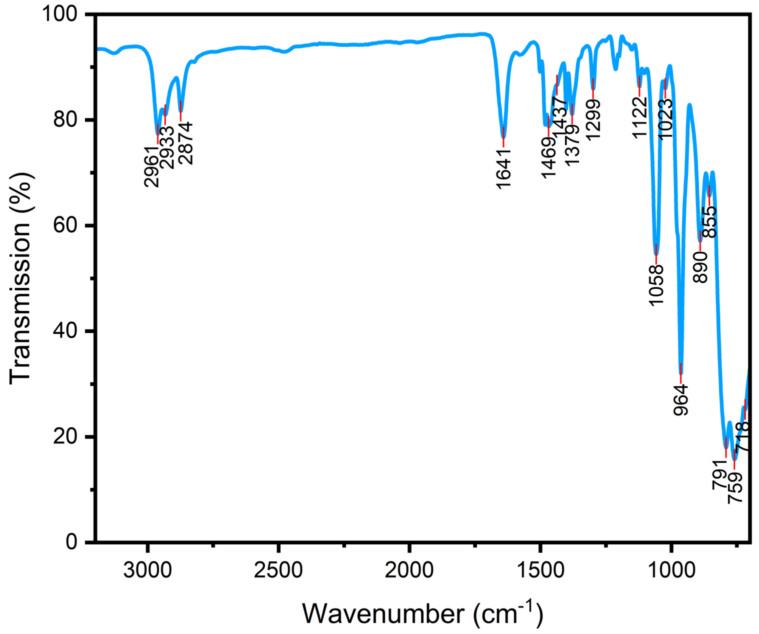

Figure S7. Infrared spectrum of **2-TpU^{V}PW$_{11}$**.

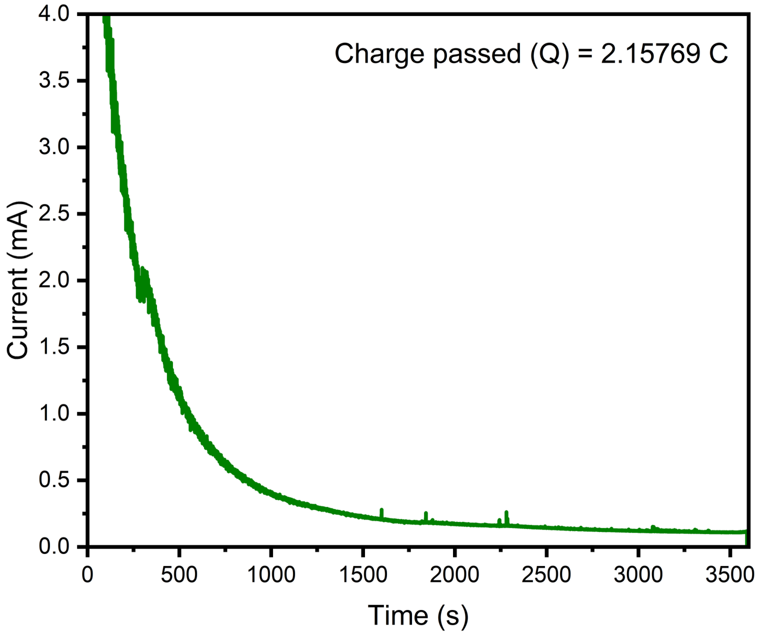

Figure S8. Bulk electrolysis of a solution of **1-TpUPW$_{11}$** (1 mM) in MeCN (100 mM TBA(PF_6)) at 1.18 V vs $Fc^{+/0}$.

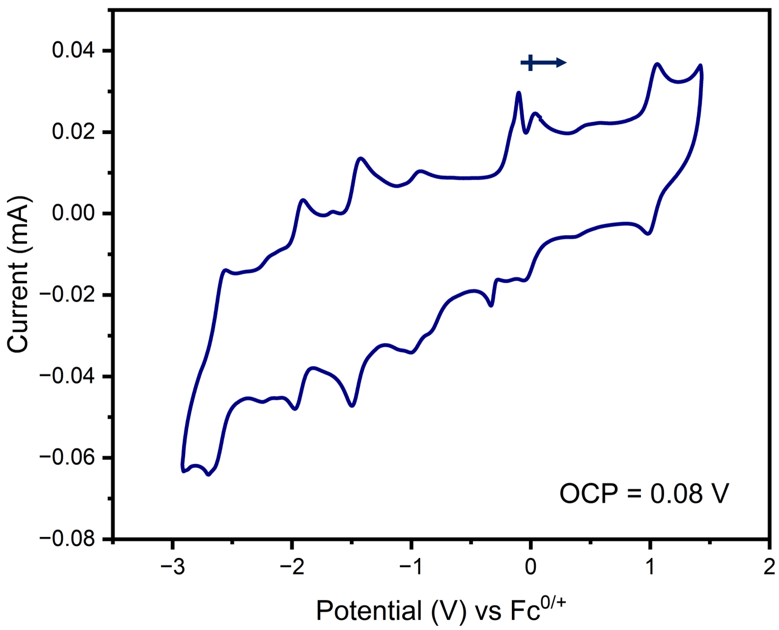

Figure S9. Post bulk electrolysis (1.18 V vs $Fc^{+/0}$) CV of **1-TpUPW$_{11}$** (1 mM) in MeCN (100 mM TBA(PF_6)).

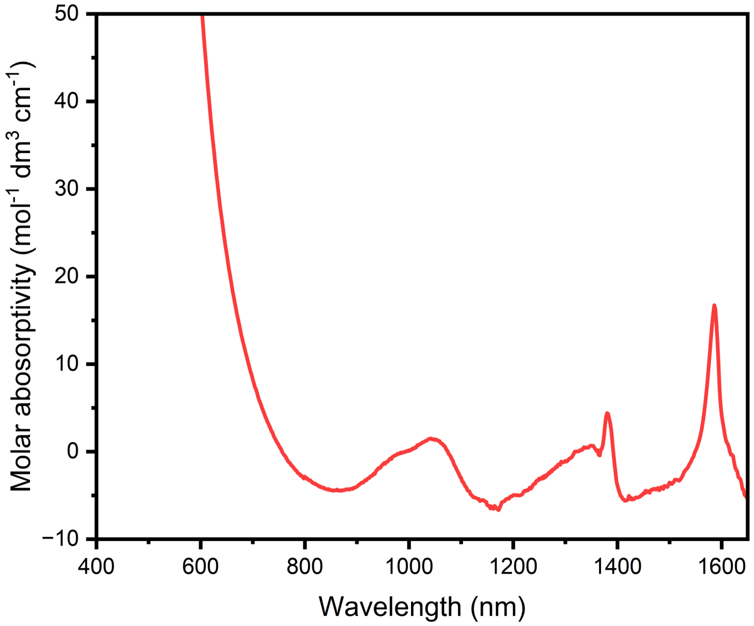

Figure S10. Post bulk electrolysis (1.18 V vs $Fc^{+/0}$) UV-Vis/NIR spectrum of **1-TpUPW$_{11}$** (1 mM) in MeCN (100 mM TBA(PF_6)). Spectrum was recorded at 21 °C.

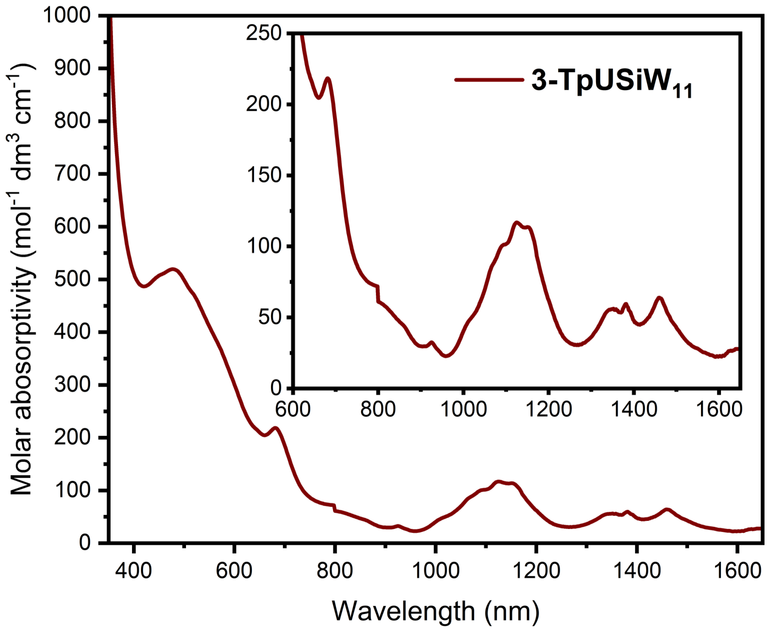

Figure S11. UV-Vis/NIR spectrum of **3-TpUSiW$_{11}$** (1 mM) in MeCN. Spectrum was recorded at 21 °C.

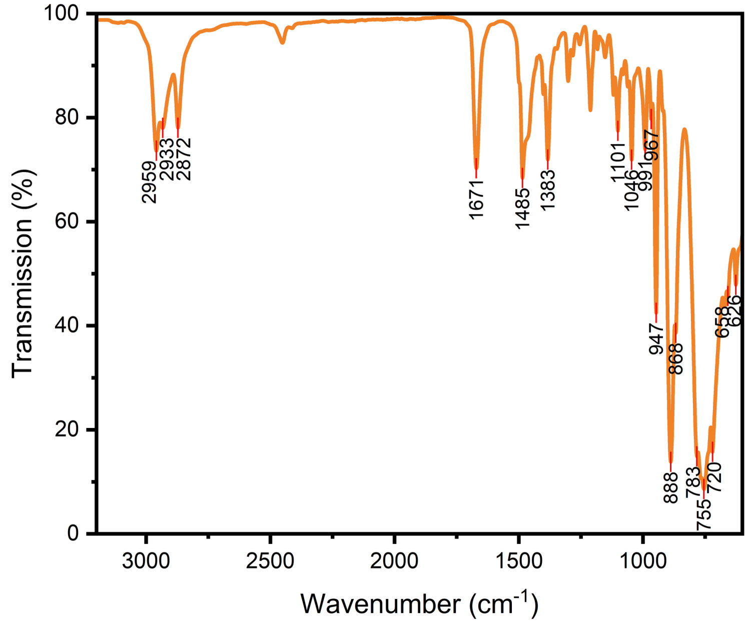

Figure S12. Infrared spectrum of **3-TpUSiW$_{11}$**.

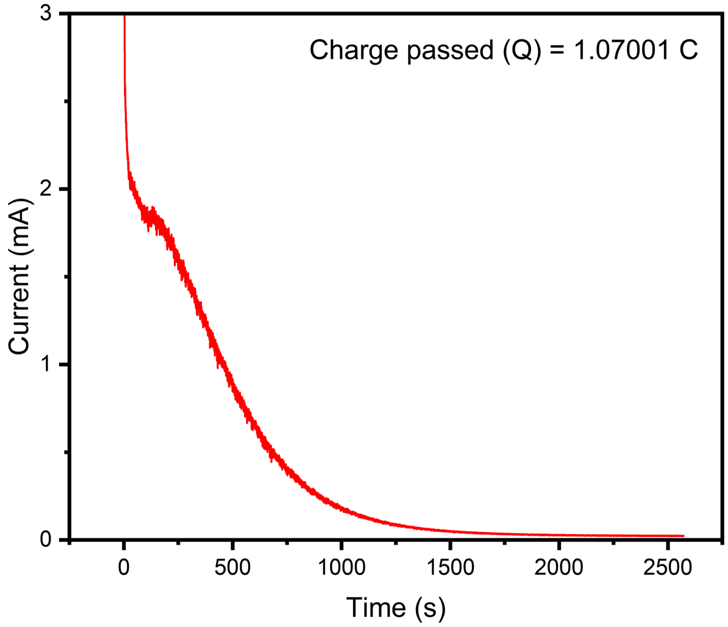

Figure S13. Bulk electrolysis of a solution of **3-TpUSiW$_{11}$** (1 mM) in MeCN (100 mM TBA(PF_6)) at −0.15 V vs $Fc^{+/0}$.

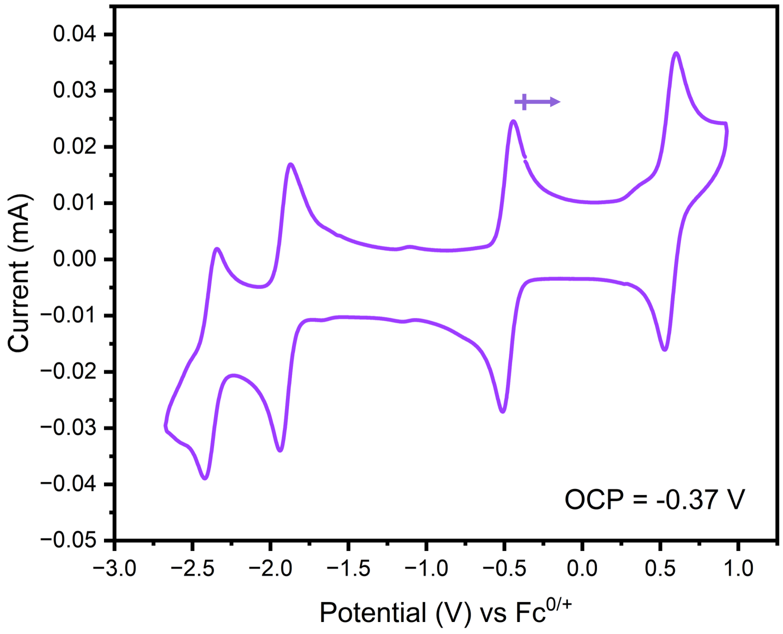

Figure S14. Post bulk electrolysis (−0.15 V vs $Fc^{+/0}$) CV of **3-TpUSiW$_{11}$** (1 mM) in MeCN (100 mM TBA(PF_6)).

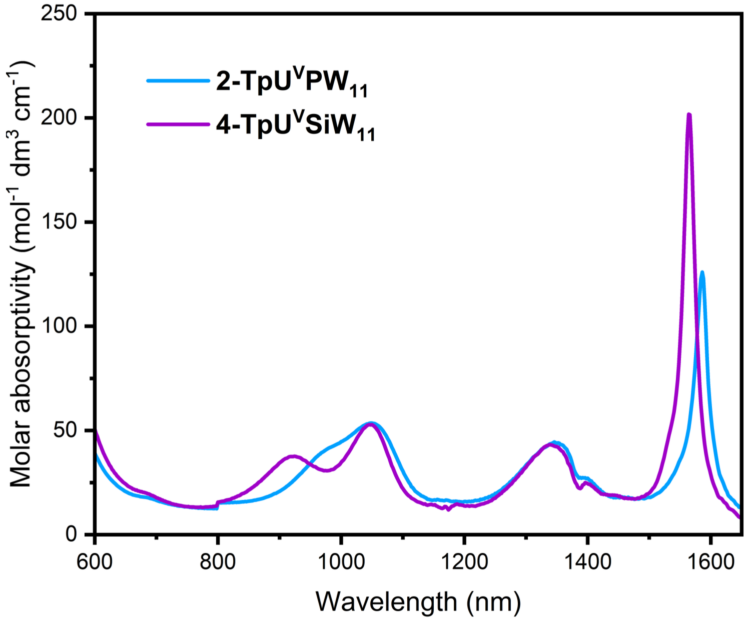

Figure S15. Comparison of the UV-Vis/NIR spectra of **2-TpU^{V}PW$_{11}$** and **4-TpU^{V}SiW$_{11}$** (both at 1 mM) in MeCN. Spectra were recorded at 21 °C.

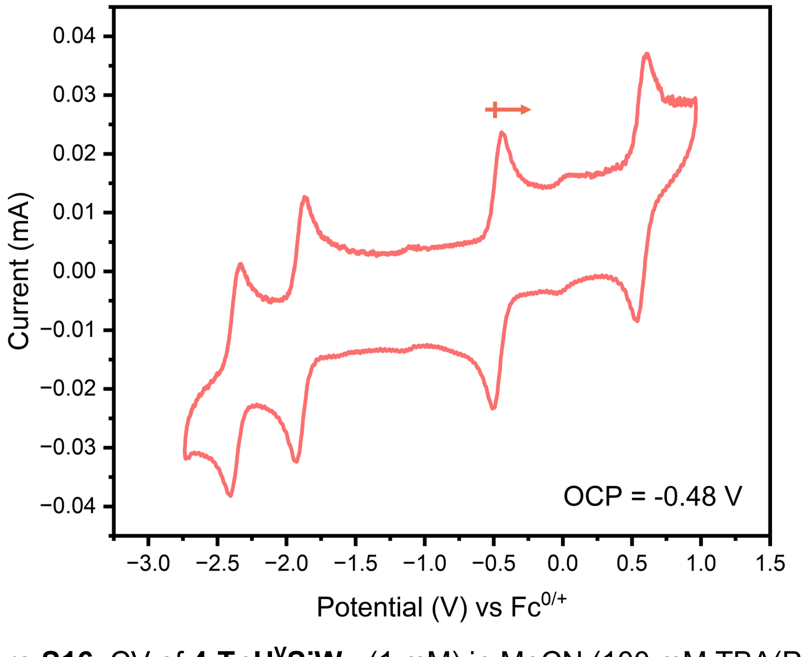

Figure S16. CV of **4-TpU^{V}SiW$_{11}$** (1 mM) in MeCN (100 mM TBA(PF$_6$)).

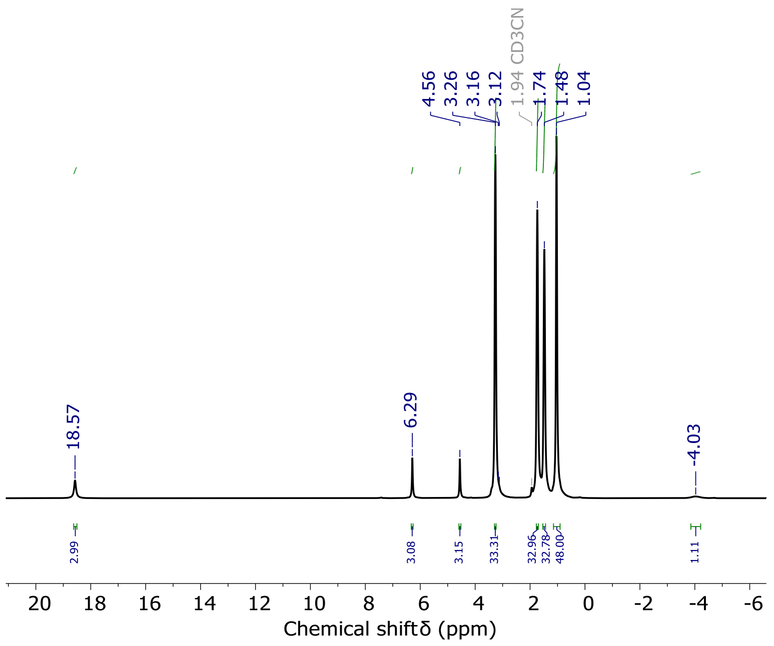

Figure S17. ^{1}H NMR (500 MHz) of **4-TpU^{V}SiW$_{11}$** in CD_3CN. Spectrum acquired at 21 °C.

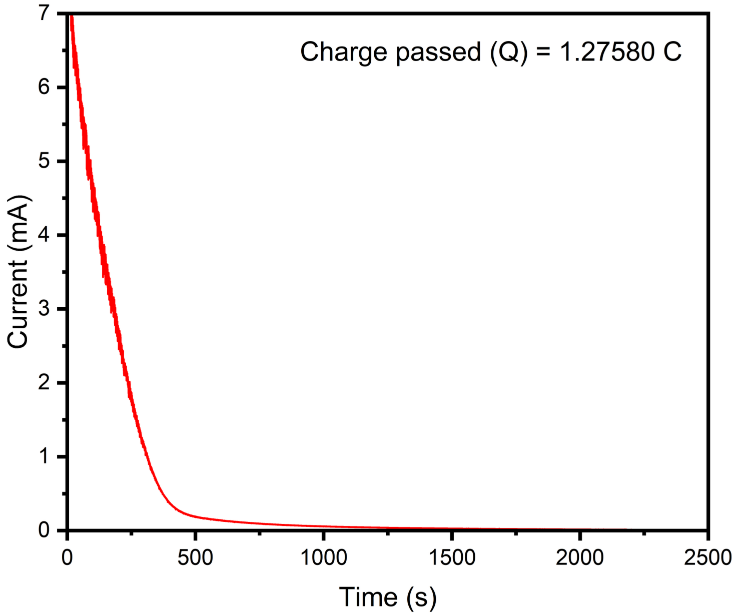

Figure S18. Bulk electrolysis of a solution of **4-TpU^{V}SiW$_{11}$** (1 mM) in MeCN (100 mM TBA(PF_6)) at 0.75 V vs $Fc^{+/0}$.

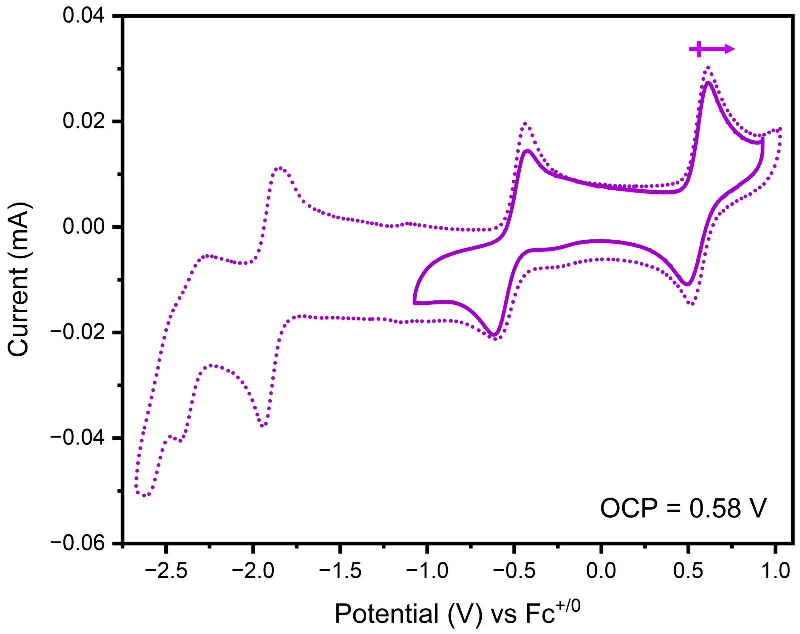

Figure S19. Post bulk electrolysis (0.75 V vs $Fc^{+/0}$) CV of **4-TpU^{V}SiW$_{11}$** (1 mM) in MeCN (100 mM TBA(PF_6)).

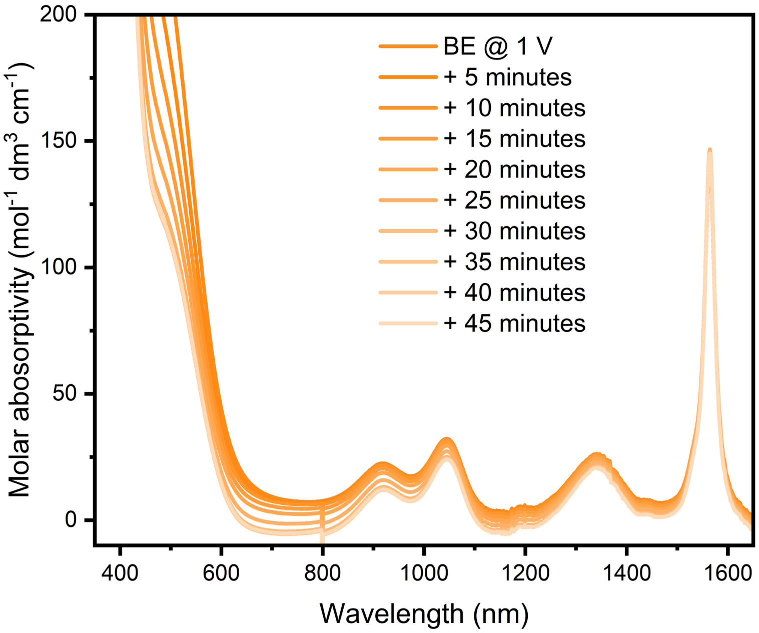

Figure S20. UV-Vis/NIR spectra of a solution of **4-TpU^{V}SiW$_{11}$** (1 mM) in MeCN over one hour at 21 °C (room temperature).

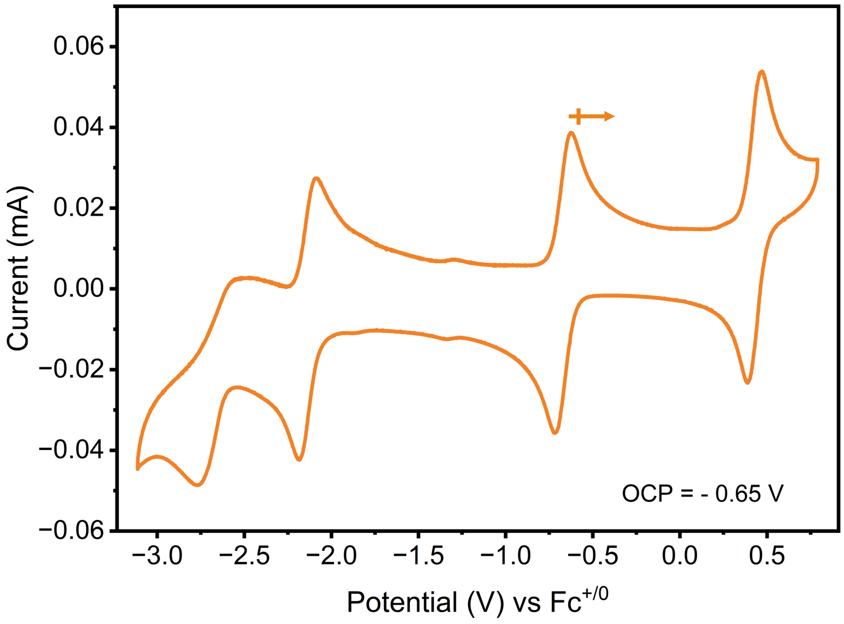

Figure S21. CV of **4-TpU^{V}SiW$_{11}$** (1 mM) in DMF (100 mM TBA(PF_6)).

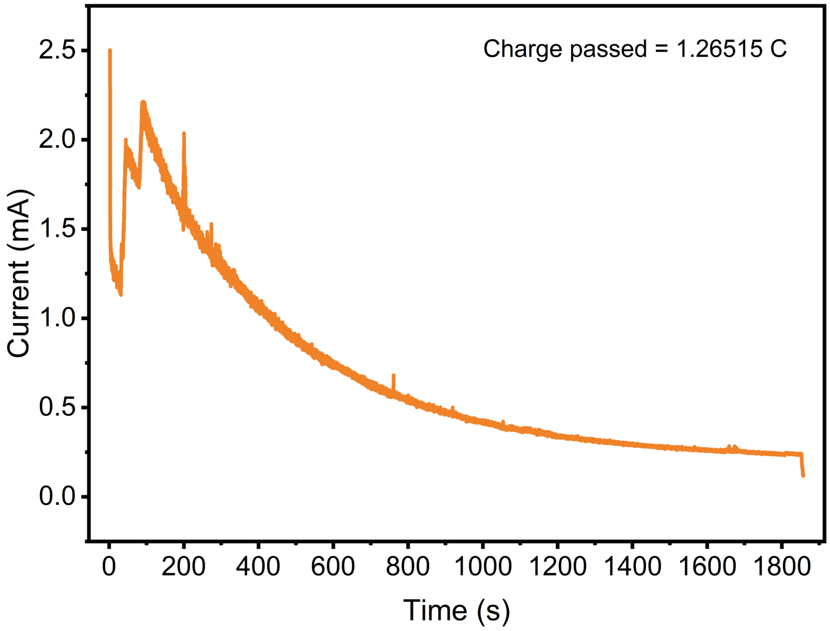

Figure S22. Bulk electrolysis of a solution of **4-TpU^{V}SiW$_{11}$** (1 mM) in DMF (100 mM TBA(PF_6)) at 0.69 V vs $Fc^{+/0}$.

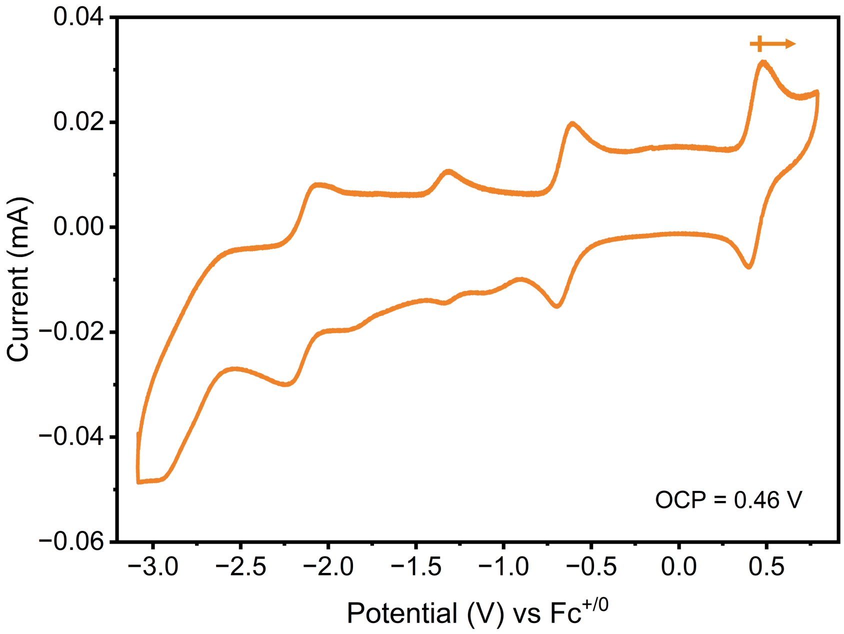

Figure S23. Post bulk electrolysis (0.69 V vs $Fc^{+/0}$) CV of **4-TpU^{V}SiW$_{11}$** (1 mM) in DMF (100 mM TBA(PF_6)).

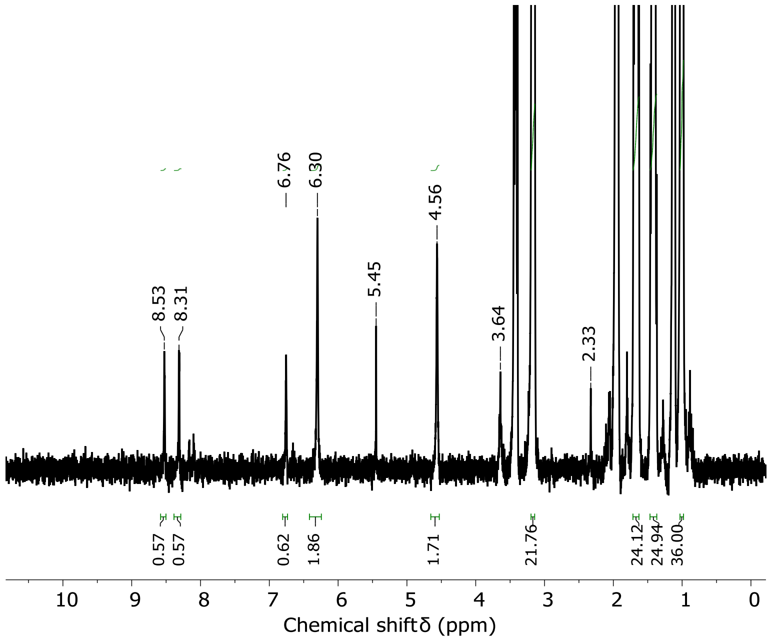

Figure S24. ^{1}H NMR (500 MHz) obtained after reaction of **4-TpU^{V}SiW$_{11}$** with 1 eq. of [N(C_6H_4Br-4)$_3$][$SbCl_6$] and work-up (see experimental section for details). The spectrum was acquired in CD_3CN at 21 °C. The peak at 6.30 ppm assigned to unreacted **4-TpU^{V}SiW$_{11}$**. The peaks at 5.45 ppm and ca. 1.1 ppm/3.4 ppm are assigned to DCM and Et_2O.

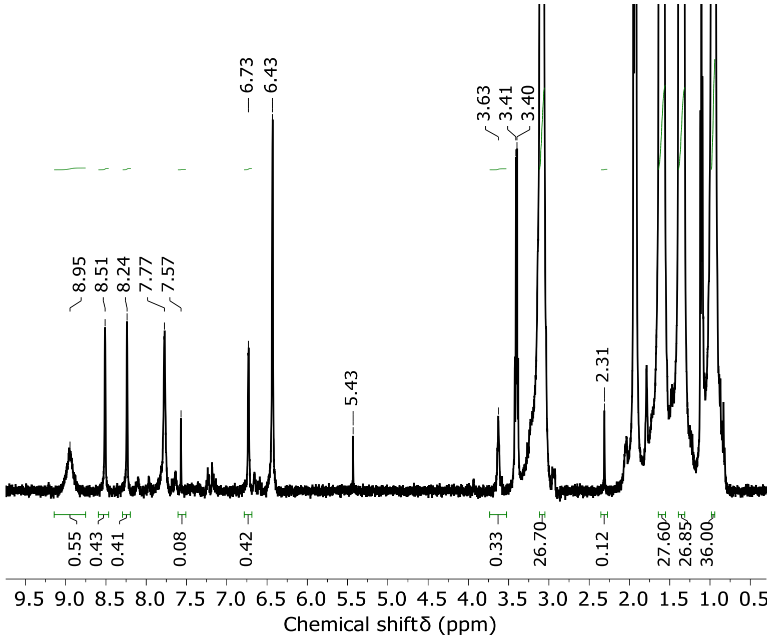

Figure S25. ^{1}H NMR (500 MHz) obtained after reaction of **4-TpU^{V}SiW$_{11}$** with 2 eq. of [N(C_6H_4Br-4)$_3$][$SbCl_6$] and work-up (see experimental section for details). The spectrum was acquired in CD_3CNat 21 °C. Peaks at ca. 1.1 and 3.4 ppm are assigned to Et_2O.

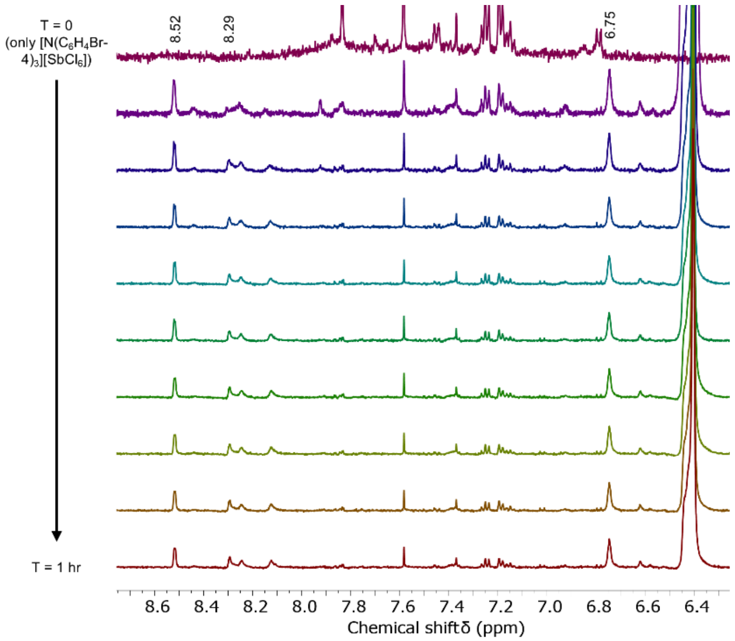

Figure S26. ^{1}H NMR (500 MHz) spectra following the reaction of **4-TpU^{V}SiW$_{11}$** with 1 eq. of [N(C_6H_4Br-4)$_3$][$SbCl_6$]. A solution of [N(C_6H_4Br-4)$_3$][$SbCl_6$] in CD_3CN was cooled to −30 °C and then treated with a solution of **4-TpU^{V}SiW$_{11}$** at −30 °C. The solution was allowed to warm to room temperature as ^{1}H NMR spectra were recorded.

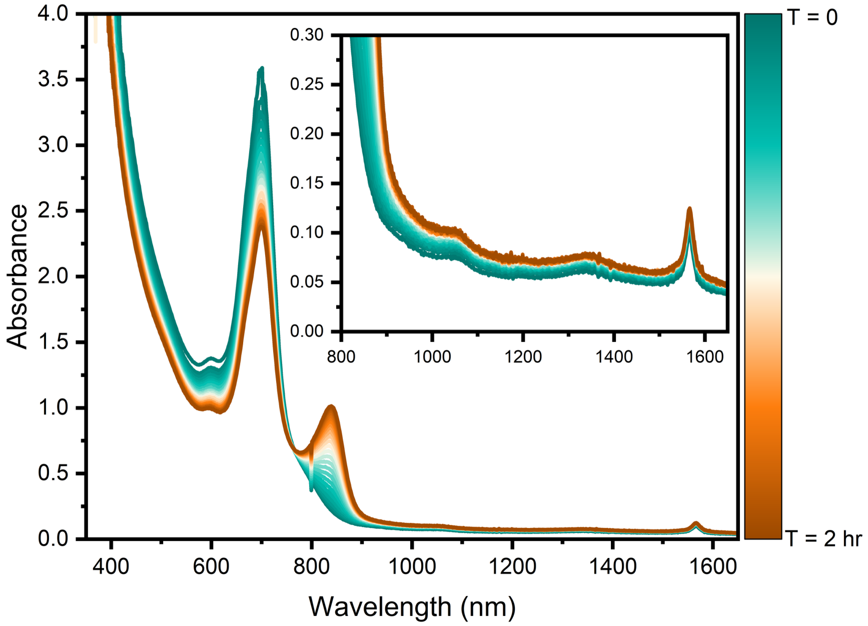

Figure S27. UV-Vis/NIR spectra following the reaction of **4-TpU^{V}SiW$_{11}$** with 1 eq. of [N(C_6H_4Br-4)$_3$][$SbCl_6$]. A solution of [N(C_6H_4Br-4)$_3$][$SbCl_6$] in MeCN was cooled to −40 °C and then treated with a solution of **4-TpU^{V}SiW$_{11}$** at −40 °C. The solution was held at −40 °C and stirred as spectra were recorded. Features associated with **4-TpU^{V}SiW$_{11}$** (i.e. f-f transitions at 1048 nm, 1340 nm, and 1564 nm) only grow after initial mixing (T = 0) showing incomplete consumption of the starting material. Also charge transfer features at <600 nm only recedes during the experiment, suggesting if any (TBA)$_3$[TpU^{VI}SiW$_{11}$O$_{39}$] forms, it decomposes during the reaction.
